# Vision techniques for anatomical structures in laparoscopic surgery: a comprehensive review

**DOI:** 10.3389/fsurg.2025.1557153

**Published:** 2025-04-14

**Authors:** Ru Zhou, Dan Wang, Hanwei Zhang, Ying Zhu, Lijun Zhang, Tianxiang Chen, Wenqiang Liao, Zi Ye

**Affiliations:** ^1^ Department of General Surgery, RuiJin Hospital LuWan Branch, Shanghai Jiaotong University School of Medicine, Shanghai, China; ^2^ Hangzhou Institute for Advanced Study, University of Chinese Academy of Sciences, Zhejiang, Hangzhou, China; ^3^ Institute of Intelligent Software, Guangzhou, Guangdong, China; ^4^ Institute of Software Chinese Academy of Sciences, Beijing, China; ^5^ School of Cyber Space and Technology, University of Science and Technology of China, Hefei, China

**Keywords:** computer vision, laparoscopic surgery, segmentation, classification, object detection

## Abstract

Laparoscopic surgery is the method of choice for numerous surgical procedures, while it confronts a lot of challenges. Computer vision exerts a vital role in addressing these challenges and has become a research hotspot, especially in the classification, segmentation, and target detection of abdominal anatomical structures. This study presents a comprehensive review of the last decade of research in this area. At first, a categorized overview of the core subtasks is presented regarding their relevance and applicability to real-world medical scenarios. Second, the dataset used in the experimental validation is statistically analyzed. Subsequently, the technical approaches and trends of classification, segmentation, and target detection tasks are explored in detail, highlighting their advantages, limitations, and practical implications. Additionally, evaluation methods for the three types of tasks are discussed. Finally, gaps in current research are identified. Meanwhile, the great potential for development in this area is emphasized.

## 1 Introduction

Laparoscopic surgery refers to an advanced, minimally invasive surgical technique. However, laparoscopic surgery faces many challenges, including limited field of view and image quality issues (1–3). This also raises the bar for surgeons, requiring enhanced spatial positioning, hand-eye coordination, and technical skills (4, 5). Computer vision (CV) has emerged as a promising tool to address these challenges, which can potentially enhance the accuracy and efficiency of laparoscopic procedures.

CV has made significant progress in its application to laparoscopic surgery through simulating the human visual system and utilizing algorithms such as image processing, machine learning, and deep learning(DL) to extract useful features from surgical images. It deals with a variety of problems associated with laparoscopic surgery (6), including improving image quality, providing real-time feedback, error detection during surgery, assisting in identifying and localizing anatomical structures. Artificial intelligence (AI)-driven approaches have been developed for addressing these critical tasks. For example, machine learning models have been employed to classify surgical maneuvers, identify surgical stages, and even predict potential complications, therefore greatly aiding in surgical planning and execution (7). These innovations have improved the accuracy and efficiency of surgery, contributed to better training and assessment of surgical skills, as well as enhanced standardization and safety in surgery. Visual tasks concentrating on the organs in the abdomen can help surgeons by guiding them visually and assisting them to find and identify structures within the body (8). For example, accurately segmenting abdominal organs to show their contours and locations can contribute to avoiding damaging vital structures during surgery, reducing surgical risks, and improving the safety and success rate of surgery (9). Automatic identification and localization of lesion areas, foreign objects, or abnormal structures can provide doctors with diagnostic basis and treatment recommendations. This can facilitate rapid diagnosis, timely and effective treatment measures, shorten operation time, and improve the success rate of surgery and the patient’s treatment experience.

Despite the advancements and the promising potential of CV in laparoscopic surgery, there is an increasing need for a comprehensive review to synthesize the existing research and guide future developments. The field is rapidly evolving, with numerous studies investigating various perspectives of CV applications in laparoscopic surgery. However, these studies usually focus on specific tasks or techniques, making it challenging for researchers and practitioners to have a clear and cohesive understanding of the overall landscape (10). A systematic review that collates and critically evaluates the current state of CV methods for anatomical structure analysis in laparoscopic images can provide valuable insights, identify existing literature gaps, and suggest future research directions. The review can also serve as a resource for developing standardized protocols and benchmarks for the evaluation of AI systems in this domain.

**Figure 1:**
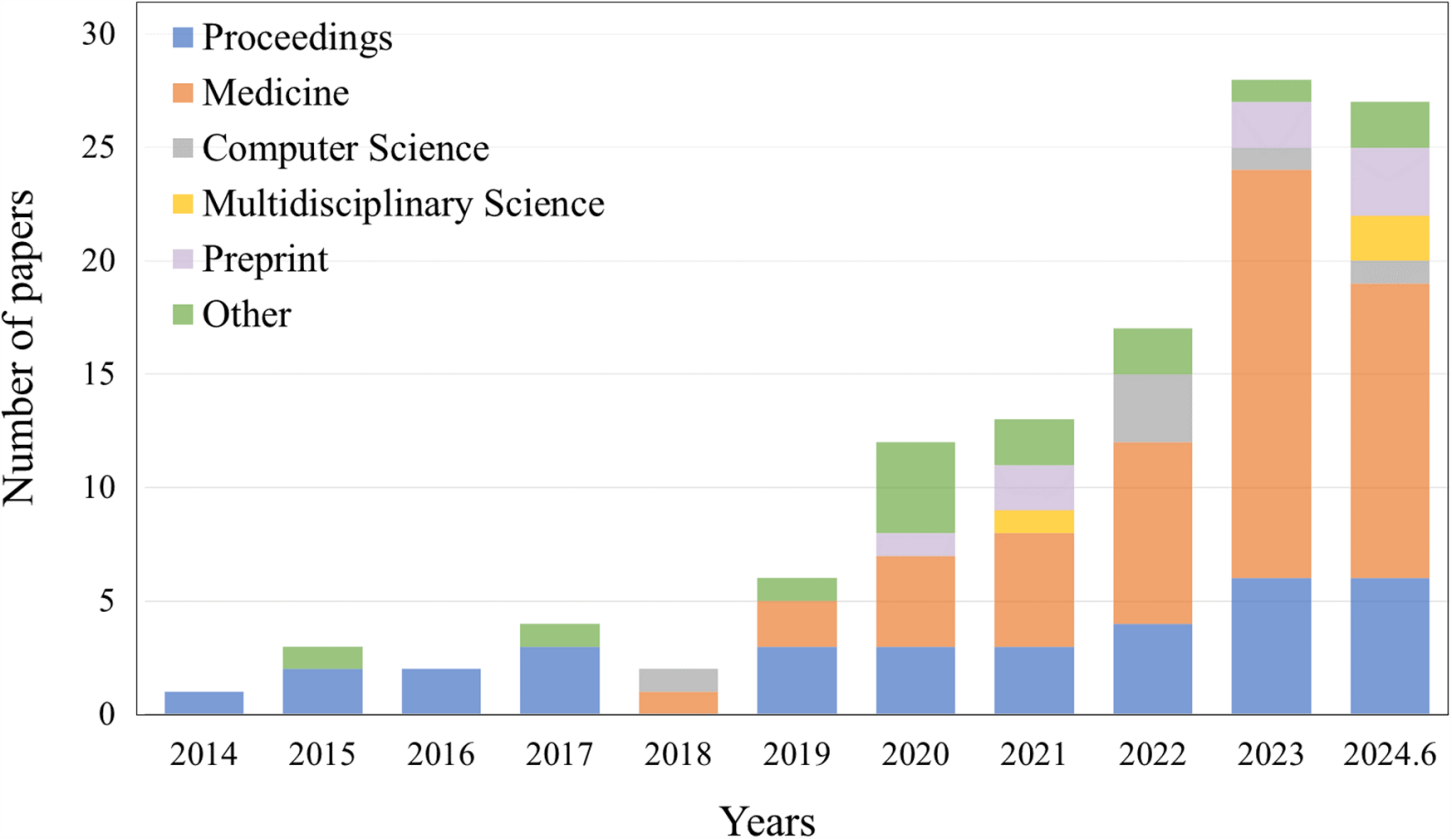
The number of papers searched on other platforms, such as conferences, different types of journals, preprint platforms, etc., according to the selection criteria, are listed by different years, ranging from 2014 to June 2024.

Currently, the segmentation, classification, and object detection tasks for abdominal organs in laparoscopy are intensively studied. We searched for references on Google Scholar. The results of the search query revealed 117 papers associated with the topic. Specific data on the sources of articles from conferences, journals, and preprints, as well as other platforms, are shown in Figure 1. The time span is from 2014 to June 2024, with the majority coming from 2023 and 2024. We specifically examined articles from IEEE, Science Direct, PubMed, and Springer, as well as the proceedings of medical imaging conferences, including MICCAI, IPMI, ISBI, RSNA, and SPIE. Figure 5a illustrates the percentage of all included studies for the three tasks of segmentation, classification, and target detection, which we will examine in detail below and suggest future research directions. Therefore, this work has the following three contributions:

• Comprehensive Survey: We conducted an exhaustive survey of the existing literature on using CV in laparoscopic surgery, systematically searching and analyzing articles on segmentation, classification, and object detection of anatomical structures.
• Task-specific categorization: We systematically categorize the core subtasks in the context of real-world healthcare scenarios, emphasizing their relevance and applicability in the clinical environment.
• Data and Metrics: We discuss the datasets commonly applied in this field, their characteristics, and the importance of using consistent and comprehensive evaluation indicators in order to facilitate fair comparison and evaluation of methods.
• Methodological Insights: We discuss the reviewed literature from task-specific and methodological perspectives, emphasizing their strengths, limitations, as well as practical implications.
• Future Directions: We identify key challenges, indicate potential research directions, and highlight the potential for future developments to advance CV use in laparoscopic surgery.

This study is structured as follows: Section 2 describes the specific healthcare scenarios and tasks covered in the selected literature. Section 3 describes in detail the datasets used in the relevant publications. Then, Section 4 provides a detailed analysis of the methods in the related literature from different perspectives, including learning strategies. Finally, Section 5 summarizes the results of this study, highlights the main findings, and provides an outlook on possible further developments in this research area.

## 2 Core tasks and application scenarios

**Figure 2:**
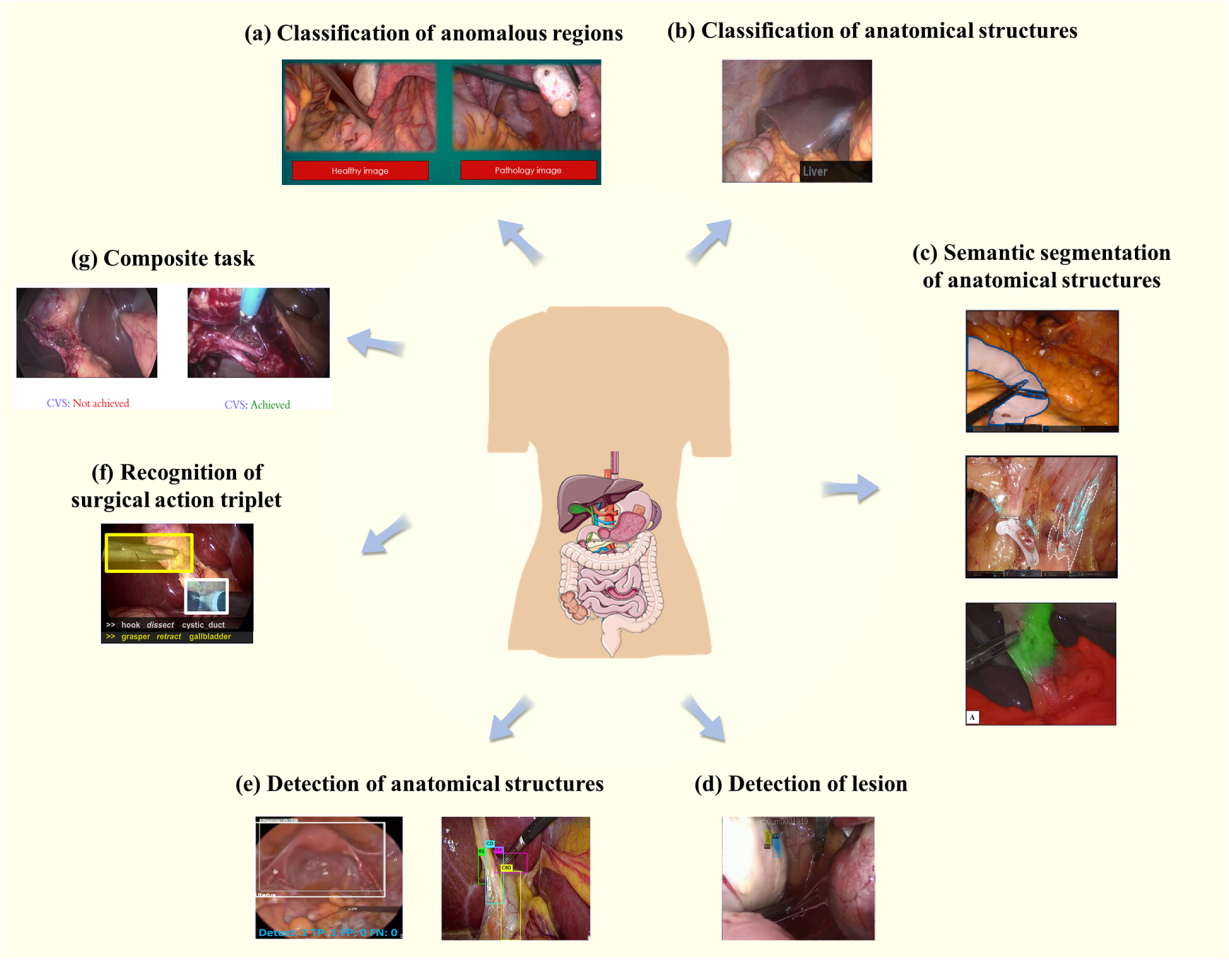
Core tasks and application scenarios. **(a)** Represents the classification of abnormal regions, while **(b)** focuses on the classification of anatomical structures. **(c)** Demonstrates semantic segmentation of anatomical structures, and **(d)** highlights lesion detection. **(e)** Shows the detection of anatomical structures, whereas **(f)** illustrates the recognition of the triad. Finally, **(g)** presents composite tasks that integrate multiple objectives.

As presented in Figure 2, seven types of core tasks are summarized in the study for classification, segmentation, and target detection of anatomical structures in laparoscopic surgical images. These sub-tasks and their application scenarios will be detailed in the following section in order to investigate their importance and application effects in the actual surgical process.

### 2.1 Classification of anomalous regions

This task is typically a binary classification task helping physicians to make faster and more accurate diagnoses and treatment plans by classifying images as normal or abnormal, as presented in Figure 2a (11). For an image I, find f such that:

(1) f(I)={normal,anomalous}

In Equation 1, normal represents the normal category and anomalous represents the abnormal category.

Common subtasks include classification of endometriosis, which is difficult because the differences between normal and pathological tissues are difficult to discern for non-specialists, the lesion sites have diversity and complexity, and there exist significant variations in visual appearance within and between people (12). Image classification techniques can significantly enhance the accuracy of diagnosing endometriosis and reduce the need for multiple procedures due to missed lesion sites. Visalaxi et al. (13) applied Resnet50 to automatically diagnose endometriosis with 91% accuracy.

### 2.2 Classification of anatomical structures

This task assigns category labels to an entire image or region of interest (ROI). Common tasks include organ categorization, i.e., categorizing different organs in an image or rating a class of organs, as presented in Figure 2b (14). Considering an image or ROI R, find a function f such that:

(2) f(R)=l

In Equation 2, $l\in L_{\text{structures}}$ and $L_{\text{structure}}$ represents the type of anatomical structures.

For example, full-resolution convolutional neural network(FrCNN) (15) is adopted for automated detection of different organs such as the uterus, ovaries, fallopian tubes. Another challenging task is grading the vascularity of the gallbladder wall, which involves scrutinizing the vascular morphology as well as the thickness, inflammatory state, and fat coverage of the gallbladder wall. Fatty infiltration or thickening of the gallbladder wall adds to the difficulty of evaluation (16). Loukas et al. (17) achieved image- and video-level classification of blood vessels in the gallbladder wall through multiple instance learning.

### 2.3 Semantic segmentation of anatomical structures

The task is aimed at assigning a category label to each pixel in an image containing common abdominal anatomical structures such as liver, kidney, gallbladder, and fat. This segmentation method goes beyond simply dividing up regions in an image to comprehend the semantic information of the various regions, i.e., the type of anatomical structure that each region represents, as presented in Figure 2c (18). Given an input image I, the objective is to find a function f such that:

(3) $$f(I)=\{L_{ij}{\}}_{i=1,j=1}^{H,W}$$

In Equation 3, $L_{ij}\in L_{\text{structure}}$ and $L_{\text{structure}}$ is the set of class labels for anatomical structures, H×W represent the dimensions of the image.

Transanal total rectal mesorectal excision (TaTME) has become the focus of recent laparoscopic research and is an important tool for treating colon and rectal cancers. LDCNet (19) can segment organs and tissues appearing in TaTME, such as the liver, gallbladder, spleen, and intestines. So that robots could help with anterior rectal resections or rectal extirpations, Kolbinger et al. (20) made a machine-learning model that can correctly name 11 types of tissues.

Compared to larger organs, tissues are representatively more dispersed, smaller, finer in granularity, and highly variable in appearance. They may blend with the surrounding anatomical region, making precise segmentation more challenging. Loose connective tissue fibers (LCTFs) are a complicated, hard-to-spot anatomical structure. Careful removal of loose connective tissue fibers improves cancer prognosis and reduces the risk of surgical complications. Kumazu et al. (21) then trained Unet to segment LCTFs. Through cutting away loose connective tissue in the rectal mesentery, Total Mesorectal Excision(TME) lowers the risk of positive circumferential margins and is a strong indicator of local recurrence (22). SwinPA-Net (23) identifies loose connective tissue in the rectal mesentery, helping to avoid damage to vital tissues such as blood vessels and nerves during surgery.

In addition, artificially defined anatomical regions are needed in specific surgical scenarios, usually areas that need to be precisely manipulated or observed during surgery. The surgeon must accurately remove the mesenteric tissue surrounding the rectum during TME. As a result, a common semantic segmentation task is identifying and labeling the anatomical lines of the rectal mesentery (24).

### 2.4 Detection of lesion

The current task aims to identify and localize possible lesion regions in an image, determining the presence of a lesion and its location and labeling it with a bounding box, as presented in Figure 2d (18). Given an image I, find f such that:

(4) $$f(I)=\{B_{i}{\}}_{i=1}^{M}$$

In Equation 4, $B_{i}$ are the coordinates of the lesion, $B_{i}=(x_{i1},y_{i1},x_{i2},y_{i2})$

Leibetseder et al. (18) applied Faster R-CNN and Mask R-CNN to find areas of endometriosis in laparoscopic gynecological videos and give confidence to those areas.

### 2.5 Detection of anatomical structures

Typically, lesion identification tasks detect relatively small and fewer targets because lesions are usually localized anomalous regions in an image. By contrast, the anatomical structure recognition task can be more complex. The task aims to automatically identify and localize different anatomical structures appearing in an image, usually involving multiple targets of different shapes and sizes, each with its own unique characteristics, as presented in Figure 2e (25, 26). For an image I, find f such that:

(5) $$f(I)=\{(B_{i},l_{i}){\}}_{i=1}^{N}$$

In Equation 5, $B_{i}$ are the bounding boxes, $B_{i}=(x_{i1},y_{i1},x_{i2},y_{i2})$ and $l_{i}$ represent the anatomical structures labels.

Boonkong et al. (25) employed DNNs to detect the uterus in laparoscopic images. Cui et al. (27) introduced a YOLOv4-based method for recognizing vas deferens images in laparoscopic inguinal hernia repair surgery. Moreover, specific critical points are common targets for detection. They may not be actual anatomical structures, but rather “judgments” made by the surgeon based on laparoscopic images. Detecting these anatomical landmarks helps the surgeon locate organ tissues and assess their morphology, location, and interrelationships (26).

### 2.6 Recognition of surgical action triplet

In 2014, Katic et al. (28) developed the surgical action triplet: !instrument, verb, target¿. This is a task that involves correctly identifying surgical instruments, actions that are being performed, and body parts that they make interactions within complicated laparoscopic videos, as presented in Figure 2f (29). The difficulty lies in the surgical instruments’ nuances, the actions’ temporal properties, and the similarity of the target organs. To be specific, identifying surgical instruments requires a detailed analysis of their heads and handles. The interaction between the instrument and the target organ needs to be taken into consideration. Secondly, the color and texture of intra-abdominal organs may be similar due to the influence of fat or connective tissue, further increasing the difficulty of identification. In addition, the identification of the triad is highly associated with the temporal information in the video. Several of the above issues lead to making recognizing surgical triplets a challenging task. CholecTriplet2021 (30) and CholecTriplet2022 challenge (31) are endoscopic vision challenges organized by MICCAI to identify surgical action triplets in laparoscopic videos.

Given an image sequence $\{I_{t}{\}}_{t=1}^{T}$, find f such that:

(6) $$f\left(\{I_{t}{\}}_{t=1}^{T}\right)=(I,A,T)$$

In Equation 6, I is the set of instruments, A refers to the set of actions, and T indicates the set of targets.

### 2.7 Composite task

In addition to the above-mentioned tasks involving only segmentation, classification, or detection, other tasks may require multiple stages, usually combining the steps of segmentation, classification, and detection, such as predicting a critical view of safety (CVS) in laparoscopic cholecystectomy (LC), as presented in Figure 2g (32).

In LC, CVS is often used as a standard operating procedure (33). CVS is the most important field to confirm the safety of the operation, and it can only be achieved if the three conditions of “access to the gallbladder by the cystic duct and the cystic artery only” are met simultaneously (34). Therefore, predicting the CVS usually involves a two-step process: (1) accurately identifying and locating the critical tissues. localization of key tissues. (2) Reasoning about the geometric relationships between the tissues and determining the quality of their exposure to determine whether the CVS criteria are met.

The TCNN model (35) is a model that segments the hepatic capsule structures first and then evaluates the CVS using the segmentation masks. In contrast to TCNN, the Murali et al. (36) trained using only bounding box annotations, outperforming several benchmark methods and scales efficiently when trained based on segmentation masks. In addition to semantic segmentation or target detection as an intermediate step, Alapatt et al. (37) also proposed a direct prediction of CVS based on self-supervised learning without prior segmentation or identification of gallbladder structures.

## 3 Public datasets

Recently, freely available datasets have exerted a central impact on developing new methods for segmentation, classification, and target detection of abdominal organs and tissues from laparoscopic images.

**Figure 3:**
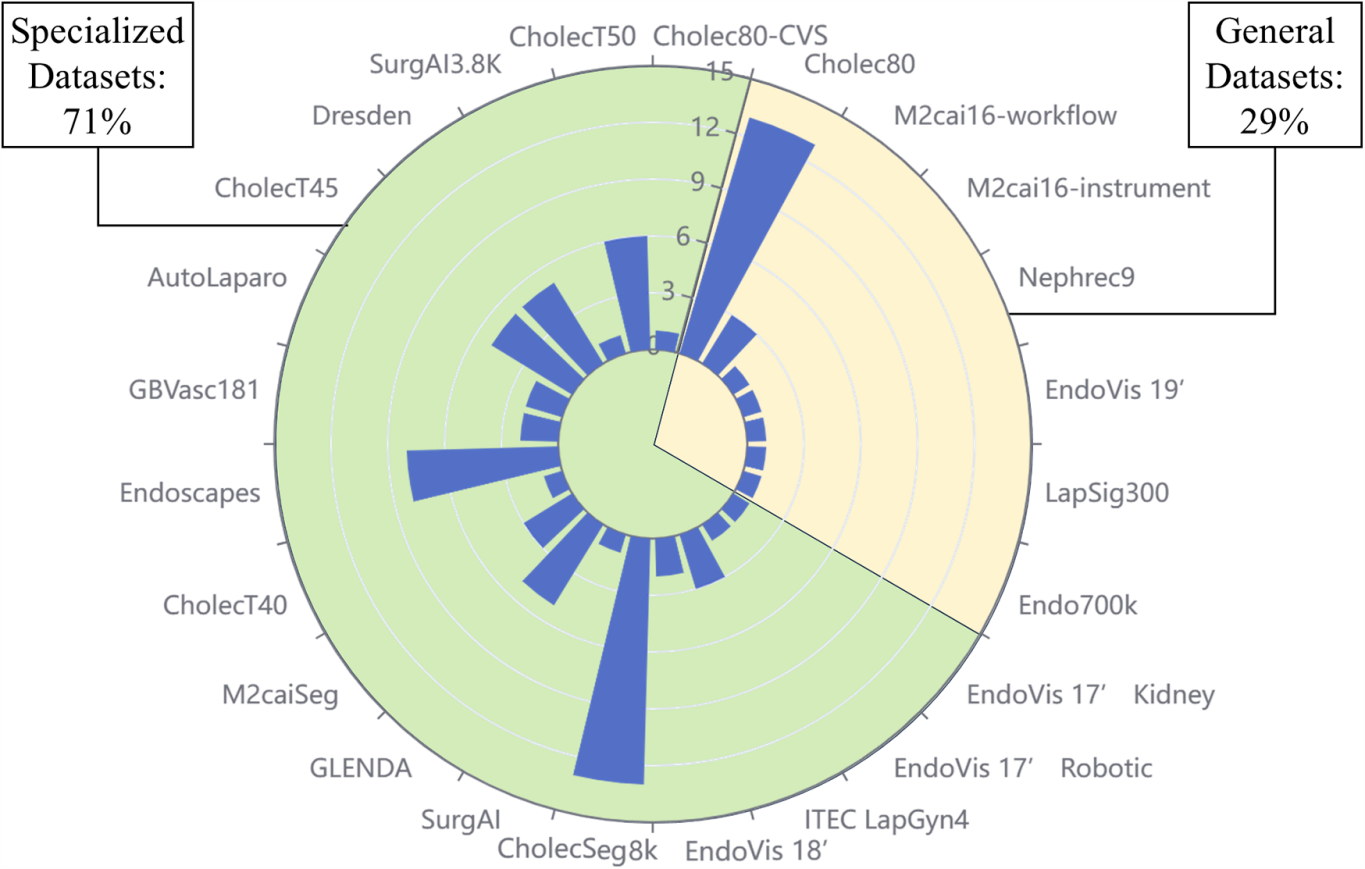
Percentage of generalized and specialized datasets and frequency of use of each dataset. The scale on the diameter indicates the number of times the dataset has been used, with a higher bar indicating a higher frequency of use. The area of the sector indicates the ratio of specialized datasets to common datasets.

With the consideration of 117 publications, it was found that totally over 90 datasets were used, categorized as public datasets, private datasets, and non-conforming datasets. “Conforming” is defined as being related to the tasks studied in this paper. Some studies involve multiple tasks, including surgical stage identification, surgical instrument segmentation, etc. The datasets which could be adopted for these additional tasks were not considered to meet the criteria. As shown in Table 1, it should be noted that the “Application” column refers to the types of tasks that can be applied to the dataset. Specifically, some of the datasets in the list are designed for such tasks, which we call specialized datasets, and the rest are generalized datasets. Figure 3 illustrates the number of times all publicly available datasets are used, as well as the percentage of generalized and specialized datasets.

**Table 1:** Public datasets.

Dataset	Year	Size	Procedure^a^	Applications	References
Cholec80	2016	80 videos	LC	Surgical phase recognition, Surgical instrument presence detection	(15, 37–40, 43, 50, 55, 84, 96–99)
M2cai16-workflow	2016	41 videos	LC	Surgical phase recognition	(39–41)
M2cai16-instrument	2017	15 videos	LC	Surgical instrument detection	(40)
EndoVis 17’ kidney Boundary detection	2017	1500 frames	PN in porcine	Kidney boundary detection	(68)
Nephrec9	2017	1262 videos	PN	Surgical phase recognition	(70)
EndoVis 17’ robotic Instrument segmentation	2017	8 videos	Porcine procedures	Surgical instrument segmentation; Tissue segmentation	(42)
ITEC LapGyn4	2018	30682 images	GLS	Surgical action recognition; Anatomical structure recognition; Action on anatomy recognition; Instrument count recognition	(15, 100, 101)
EndoVis 18’ sub-challenge	2018	30+ videos	CS	Surgical instrument segmentation; Organ segmentation	(42, 55)
EndoVis 19’ surgical Workflow and Skill analysis	2019	30+ videos	LC	Surgical workflow and skill analysis	(102)
CholecSeg8k	2020	8080 frames	LC	Anatomical structure segmentation; Surgical instrument segmentation	(44, 50, 67, 71, 76, 77, 79, 81, 99, 103–106)
SurgAI	2020	461 images	GLS	Anatomical structure segmentation; Surgical instrument segmentation	(10)
GLENDA	2020	25682 frames	GLS	Endometriosis classification; Endometriosis detection	(11, 13, 18, 47, 93)
M2caiSeg	2020	307 images	Other	Anatomical structure segmentation; Surgical instrument segmentation	(75, 99, 104)
CholecT40	2020	40 videos	LC	Surgical action triplet recognition; Surgical action triplet detection/localization; Surgical instrument presence detection; Surgical instrument detection/localization; Surgical action/verb recognition; Surgical target recognition; Surgical phase recognition	(29)
LapSig300	2020	300 videos	CS	Surgical phase recognition; Surgical action recognition	(86)
Endoscapes dataset	2021	201 videos	Other	Surgical scene segmentation; Object detection; Critical view of safety assessment	(35–37, 45, 50, 55, 55, 84)
GBVasc181	2021	181 images	LC	Gallbladder wall vascularity classification	(16, 17)
AutoLaparo	2022	21 videos	LC	Surgical workflow recognition; Laparoscope motion prediction; Instrument and key anatomy segmentation	(37, 63)
CholecT45	2022	45 videos	LC	Surgical action triplet recognition; Surgical action triplet detection/localization; Surgical instrument presence detection; Surgical instrument detection/localization; Surgical action/verb recognition; Surgical target recognition; Surgical phase recognition	(31, 32, 43, 60, 107)
Dresden surgical Anatomy dataset	2023	13195 images	Proctoco-lectomy	Anatomical structure segmentation	(9, 20, 78, 90, 108)
SurgAI3.8K	2023	3800 images	GLS	Anatomical structure segmentation	(65)
CholecT50	2023	50 videos	LC	Same as CholecT45	(31, 58, 59, 84)
Endo700k	2023	700,000+ images	Other	Label not provided	(43)
Cholec80-CVS	2023	80 videos	LC	CVS recognition	(46)

^a^For the “Procedure” column, “Other” means involving multiple types of surgery. LC, laparoscopic cholecystectomy; GLS, gynecologic laparoscopic surgeries; PN, partial nephrectomy; CS, colorectal surgery.

We have selected six of the most extensively used public datasets and categorized them into generic and specialized, which are explored separately in the following subsections.

### 3.1 General datasets

Generic datasets often lack specific labeling or annotations for the visual tasks studied in this paper, therefore requiring additional annotation work. These datasets are invaluable due to their large number and variety of images, and provide a wide base for all types of research and development of AI applications in laparoscopic surgery.

#### 3.1.1 Cholec80

The Cholec80 dataset (38) contains 80 LC videos. Each of these frames is labeled with a surgical stage and tool presence, which can be used to test the performance of tool presence detection and stage identification methods. In addition, the dataset can be used for a variety of other applications including automated surgical video database indexing, real-time surgical procedure monitoring, and optimizing operating room staffing.

In addition to Cholec80, there are several datasets from surgical flow recognition, instrument segmentation challenges, e.g., M2cai16-workflow challenge (39–41) and EndoVis 17’ Robotic Instrument Segmentation sub-challenge (42). Moreover, these datasets were also used in the study by re-labeling.

#### 3.1.2 Endo700k

Due to the scarcity of publicly available labeled data and the acquisition and labeling process complexity, Endo700k (43) is aimed at alleviating these problems through large-scale self-supervised pre-training. Endo700k is consisted of nine publicly available endoscopic datasets merged together, containing over 700,000 unlabeled images involving nine types of minimally invasive surgeries, including prostatectomy, cholecystectomy, and gastrectomy. It enables researchers to fine-tune models on large amounts of unlabeled data.

### 3.2 Specialized datasets

#### 3.2.1 CholecSeg8k

The CholecSeg8k dataset (44) is based on the Cholec80 dataset, where 17 video clips were carefully selected from 80 videos of cholecystectomy surgeries, and 8,080 image frames were extracted from them for pixel-level annotation. These images include different stages and scenarios of cholecystectomy surgeries. The CholecSeg8k dataset is finely labeled, with each image being labeled for 13 critical categories.

#### 3.2.2 Endoscapes dataset

The endoscape dataset (45) contains 201 LC videos in which frames are sparsely but regularly labeled with segmentation masks, bounding boxes, and CVS assessments. This dataset can be used for machine learning tasks, including object detection, instance segmentation, CVS prediction, and diverse experiments such as hybrid supervised, semi-supervised, and temporal modeling. The Endoscapes dataset provides four subsets covering different annotation types and data. In addition, comprehensive performance benchmarks are available, providing evaluation criteria for research.

In addition to Endoscapes, Rios et al. (46) annotated all videos in Cholec80 based on the CVS standard, which can also be applied in studies of CVS prediction.

#### 3.2.3 GLENDA

GLENDA (Gynecologic Laparoscopic Endometriosis Dataset) is a dataset identifying endometriosis (47). The dataset contains over 350 labeled images of endometriosis lesions covering the four pathologic endometriosis categories and non-pathologic control example images from more than 100 gynecologic laparoscopic procedures. Tasks including binary classification (endometriosis) and detection/localization are supported.

#### 3.2.4 CholecT50

CholecT50 (31) is a dataset that identifies action triples for laparoscopic cholecystectomy surgery. The dataset contains 50 video clips documenting laparoscopic cholecystectomy procedures, with 100 categories of surgical action triplets being labeled in detail. These annotations contained approximately 151,000 triad instances, including 6 instrument categories, 10 action verb categories, and 15 target categories.

Cholect45 and Cholect40 are two subsets of Cholect50, containing videos of 45 and 40 surgical procedures, which are also annotated with triad information in the form of [instrument, verb, goal].

#### 3.2.5 Dresden surgical anatomy dataset

The Dresden Surgical Anatomy Dataset (DSAD) (9) concentrates on solving the problem of semantic segmentation of abdominal organs.

The dataset provides totally 13,195 laparoscopic images utilizing videos of robot-assisted rectal resections and contains semantic segmentation of eight abdominal organs, the abdominal wall, and two vascular structures. Each image provides a weak annotation of the presence of the organ, providing researchers with various applications of the data.

## 4 Methodology and technical strategy

**Figure 4:**
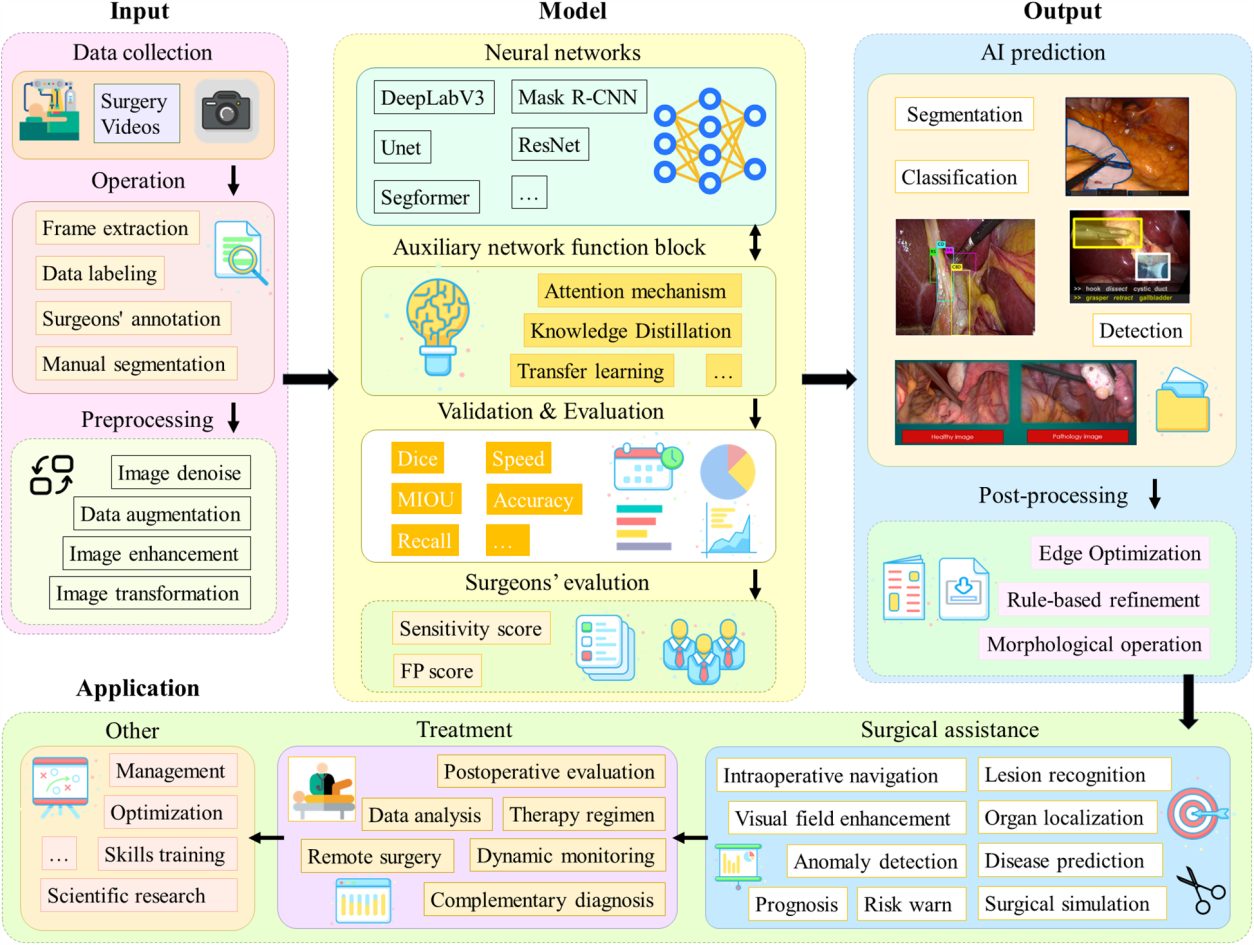
A generalized process for applying DL models to laparoscopic medical scenarios. The first part is the data acquisition and preprocessing steps; the second part is the model architecture and the model evaluation, which is the main discussion in this chapter; the third part is the model output and post-processing operations; and the fourth part is the actual application scenarios.

Most of the studies in the literature collected in this paper have applied DL methods. Figure 4 shows a generic process, containing four main modules: input, model, output and application.

Firstly, videos of laparoscopic surgical need to be collected. In order to process long video data, operations such as frame lifting are usually required. Next, segmentation masks, category labels, bounding boxes, etc. need to be labeled by the surgeon. Subsequently, the data are preprocessed aiming to improve robustness of the model. For different tasks, appropriate network architectures are selected in line with their characteristics, such as Unet and DeeplabV3, and some technical improvement modules, such as the attention mechanism, may be added. It is also possible to adopt different learning paradigms or design entirely new network architectures. Afterwards, the model performance is evaluated. Also, a team of surgeons will perform a qualitative assessment. After the model outputs the results, post-processing operations are usually needed to further enhance the quality of the results. Final result can be applied in different practical scenarios, such as lesion localization and organ identification. These data can also be used for surgical skills training as well as scientific research to advance the field of medicine and artificial intelligence.

**Figure 5:**
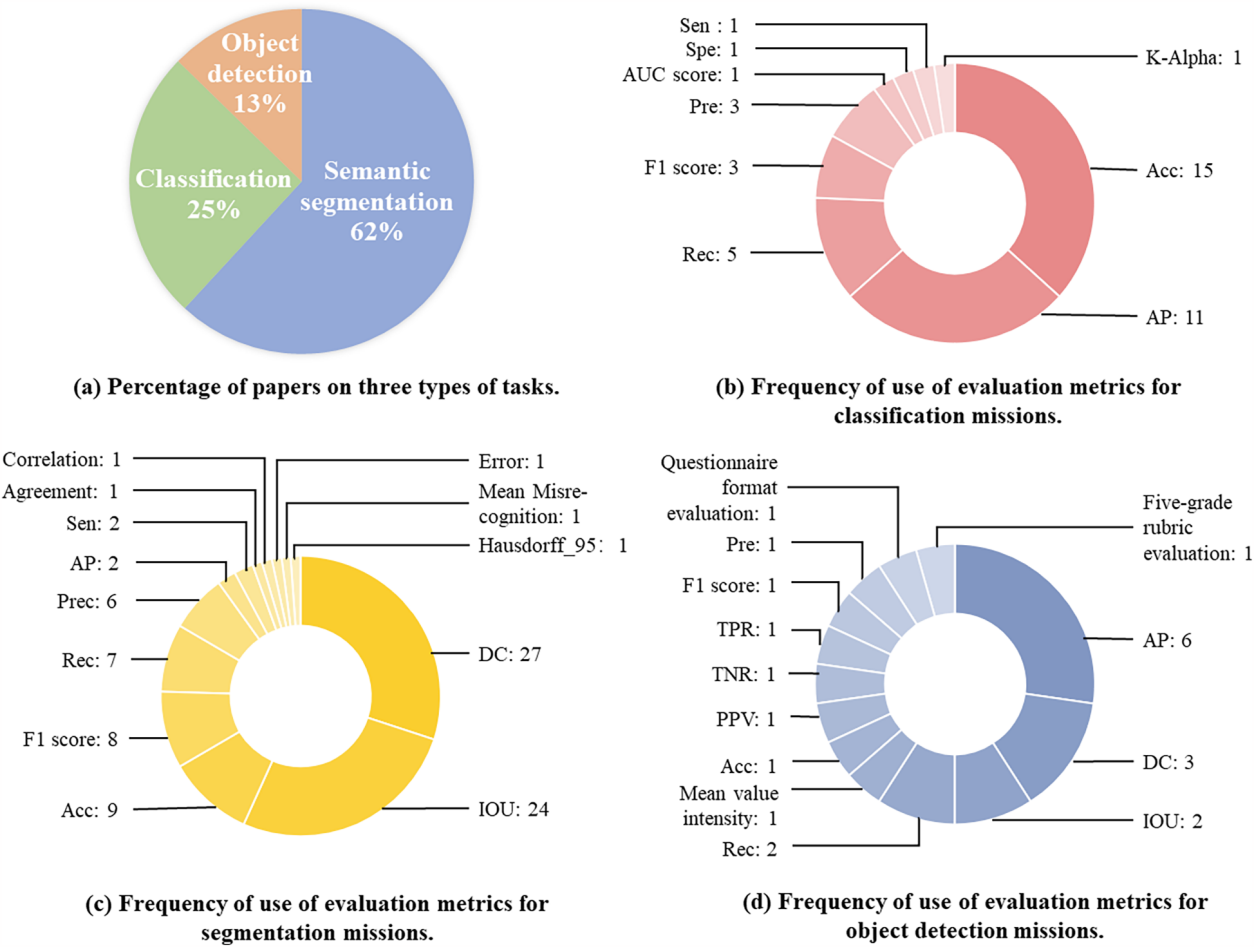
Proportion of papers across the three assignment categories and their corresponding evaluation metrics. This figure presents an analysis of research distribution and evaluation metrics in laparoscopic image analysis. **(a)** Displays the percentage of papers focusing on three types of tasks. **(b)** Illustrates the frequency of evaluation metrics used for classification tasks. **(c)** Highlights the frequency of evaluation metrics applied in segmentation tasks. **(d)** Shows the frequency of evaluation metrics employed for object detection tasks.

As presented in Figure 5, 62% of the included studies are focused on segmentation tasks (73 articles), 25% on classification tasks (32 articles), and 13% on target detection tasks (15 articles). These tasks are detailed. Next, we will analyze and detail the methodological techniques, model architecture, and strategy evaluation for each of these three types of tasks.

### 4.1 Classification methods

This section deals exclusively with publications concentrating on classification methods, where 32 contributions were identified, as presented in Table 2. They will be characterized and logically grouped according to different characteristics in the following. At first, developments are organized according to learning strategies: supervised and not supervised learning. Then, recent developments in multi-task-based learning are described. Finally, an analysis of evaluation metrics for classification methods.

**Table 2:** Classification methods.

References	Year	Learning strategy^a^	Target^c^	Model	Dataset^b^
(15)	2024	SL	Uterus, ovary, liver, colon, etc.	Improved K-means clustering+LBP+AS+Improved cuckoo search optimization+FrCNN	ITEC LapGyn4, Cholec80
(54)	2024	SL	CVS	LG-CVS+Domain Generalization+Disentangled Learning+Latent Graph+GNNs	Endoscapes
(54)	2024	WSL	Stomach, uterus, etc.	ChatGPT 4.0+SceneXplain Plugin	Proprietary
(56)	2023	SL	Triplet	Vision-Language Models+GNNs+Spatio-Temporal Reasoning Network+Visual-Semantic Reasoning Network	CholecT50
(55)	2023	SL	Anatomical structures	SurgicalGPT: GPT2+Language-Vision-GPT+Transformer	Cholec80, EndoVis18
(48)	2023	SL	Areas related to blood vessels	Temporal Label Smoothing+Classification using BNNs+Uncertainty guided temporal filtering	Proprietary
(11)	2023	SL	Endometriosis	ResNet50+Transfer learning	GLENDA
(36)	2023	WSL	CVS	LatentGraph-CVS: Graphical representations containing semantic information about anatomical structures+GNNs	Endoscapes
(37)	2023	USL	CVS	Large-scale self-supervised pretraining+Downstream task fine-tuning: MoCo v2(ResNet50)+TeCNO	AutoLaparo, Cholec80, Endoscapes
(50)	2023	SL	CVS	GNNs+Potential spatio-temporal maps+Per-frame graph prediction+Temporal edge creation	Endoscapes; Cholec80; CholecSeg8k
(59)	2023	SL	Triplet	ResNet50+LSTM+Multi-Label Mutual Channel Loss	CholecT50
(107)	2023	SL	Triplet	Temporal Attention Module+CAGTAM+Encoder-Decoder+ResNet18+CAM	CholecT45
(60)	2023	SL	Triplet	Swin Transfomer+Self-distillation+Multi-task Learning+Ensembling	CholecT45
(61)	2023	SL	Triplet	ResNet50+MCIT-IG(Transformer)+Interaction-Graph(GNNs)+Mixed Supervision	CholecT50
(43)	2023	SL	Triplet	EndoViT(Transformer)+Encoder-Decoder+Transfer learning	Endo700k, Cholec80, CholecT45
(32)	2023	SL	Triplet, CVS, gallbladder inflammation	ConceptNet(GNNs)+Knowledge Graph	CholecT45
(49)	2022	SL	CVS	DeepCVS: Multi-Stage Learning+DeepLabV3 (Xception65)+Multi-Label Classification Networks	Proprietary
(58)	2022	SL	Triplet	ResNet18+CAGAM+MHMA+Encoder-Decoder	CholecT50
(109)	2021	SL	Gallbladder inflammation	ResNet50+Multilevel Bayesian regression models	Proprietary
(110)	2021	SL	CVS	EndoDigest(DNNs)	Proprietary
(13)	2021	SL	Endometriosis	ResNet50+Transfer learning	GLENDA
(17)	2021	USL	GB wall	Multiple-instance learning+Variational Bayesian Gaussian Mixture Models+SVM	GBVasc181
(111)	2020	SL	Unqualifed, pharynx etc.	ResNet50+Inceptionv3+vgg11-bn+vgg16-bn+DenseNet121 +Transfer learning	Proprietary
(16)	2020	SL	GB wall	VGG+ResNet+SVM	GBVasc181
(96)	2020	SL	GB wall	K-Means+SVM+Naïve Bayes+CNNs	Cholec80
(112)	2020	SL	Gallbladder	CNNs	Proprietary
(41)	2020	USL	Abdominal wall, fat tissue etc.	Multi-Instance Multi-Label Learning+Variational Bayesian gaussian mixture models	M2cai16-workflow
(29)	2020	SL	Triplet	MTL+Class Activation Guide+3D Interaction Space	CholecT40
(14)	2018	SL	Uterus, ovaries, liver, colon etc.	AlexNet+GoogLeNet+SVM	Proprietary
(113)	2018	SL	Six porcine tissues: liver, spleen etc.	Hyperpixel classification strategy based on texture and reflectivity information	Proprietary
(114)	2017	SL	Ureteral etc.	GoogLeNet	Proprietary
(115)	2017	SL	Organ tissue	Multi-spectral texture analysis	Proprietary

^a^For the “Learning Strategy” column. SL, supervised learning; WSL, weakly supervised learning.

^b^For the “Dataset” column, “Proprietary” means that the dataset is not publicly available.

^c^Target may involve surgical instruments, etc. Only anatomical structures are listed here.

#### 4.1.1 Supervised learning

**Figure 6:**
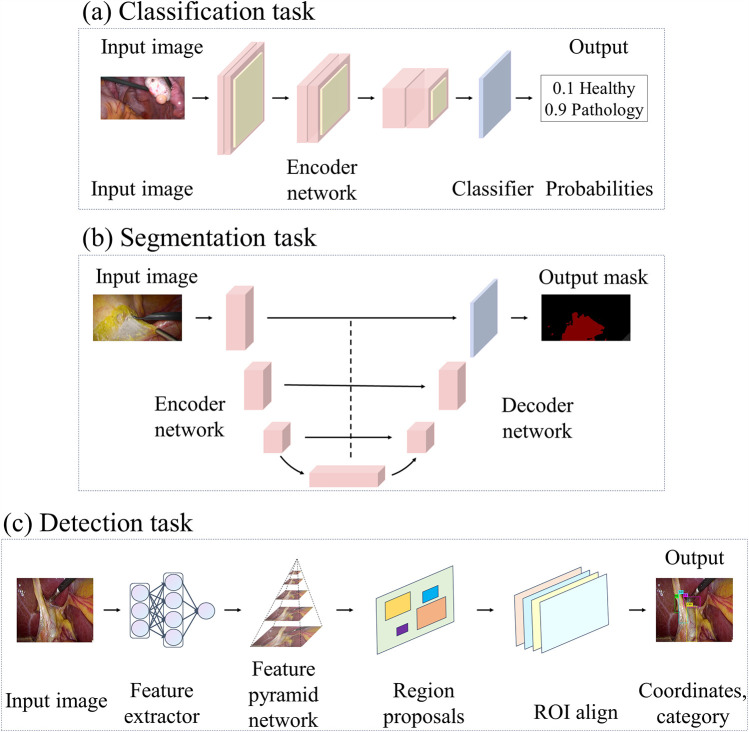
This figure illustrates the deep learning architectures employed in various laparoscopic image analysis tasks: **(a)** architectures used for classification tasks, **(b)** architectures applied in segmentation tasks, and **(c)** architectures designed for detection tasks.

Network architectures like the one shown in Figure 6a are usually used in supervised learning methods for classification tasks. The encoder can be a CNN, extracting features with semantic information from the original image through a series of convolutional layers, pooling layers, and other operations. These features retain key information in the image, including organ shape, and texture, and reduce the dimensionality of the data. Classifiers typically have one or more fully connected layers, where the output represents a probability distribution for each category and the one with the highest probability is selected as the classification result.

Practically, ResNet50 is often used as an Encoder network through methods such as transfer learning. Visalaxi et al. (13) and Nifora et al. (11) utilized ResNet50 to classify laparoscopic images to assist in the diagnosis of endometriosis, achieving 90% and over 95% accuracy rates respectively. It is of note that the latter used a larger dataset. For anatomical structure classification, Hayashi et al. (48) introduced a timestamp smoothing technique followed by classification using a Bayesian neural network. Finally, uncertainty-guided temporal filtering based on uncertainty modifies the results with high uncertainty. In addition, Konduri et al. (15) used an improved K-mean algorithm for clustering, and extracted texture and spectral features using local binary patterns and averaged spectra. Feature selection was optimized based on an improved Cuckoo search optimization algorithm. The final classification was performed through a FrCNN, and the results indicated that the method achieved more than 99% accuracy in organ classification.

Furthermore, for CVS prediction, DeepCVS (49) is a two-stage model firstly using Deeplab v3+ with Xception 65 as the backbone of a segmentation network to identify anatomical structures. Then, a multi-label classification network was then utilized to evaluate the achievement of the CVS criterion. Murali et al. (50) proposed to encode surgical videos as potential spatio-temporal maps representing anatomical structures and instruments and their properties evolving over time. A pre-trained model is first used to predict the graph for each frame. Then, the temporal edges are added between nodes based on spatial consistency and visual and semantic similarity. The article introduces a graph editing module utilizing a priori knowledge and temporal consistency to correct errors in the graphs, which can therefore improve the performance of downstream tasks. The experimental results of this article are superior to DeepCVS (49).

In recent years, the development of Large-scale language modeling(LLM) has been very rapid (51), and Vision-Language Models (VLM) have shown unprecedented potential for understanding complex surgical scenarios (52, 53). Hirides et al. (54) analyzed 100 laparoscopic surgical images directly using ChatGPT4 and its image recognition plugin SceneXplain, and their results performed well for the task of recognizing anatomical structures. SurgicalGPT (55) is an end-to-end trained VLM of gpt for visual question-answering tasks in surgical scenarios. The model extends the GPT2 model to include visual input and introduces a feature extractor and visual token embedding. By ordering word tokens before visual tokens, the model mimics the way humans think about understanding questions, and thus better infers answers based on images. Experimental results show that SurgicalGPT performs well in anatomical structure classification. Chain-of-Look (56) Prompting is an end-to-end surgical triad recognition method. The method decomposes the task into interpretable steps by constructing a series of video reasoning processes and utilizes a large-scale VLM for visual cue generation. In addition, the article introduces a verb-centric modeling scheme to emphasize the core semantic information of surgical actions. The method achieves optimal performance on the CholecT50 dataset.

#### 4.1.2 Unsupervised learning

Unsupervised learning methods are mainly applied to the CVS prediction task. These methods can effectively lower the dependence on a large amount of manually annotated data and contribute to improving the generalization ability and robustness of the model in different surgical scenarios.

Murali et al. (36) used bounding box annotations to train CVS prediction models. During the first stage, key anatomical structures are identified and used as nodes of the graph. Next, the relationships between nodes are predicted to form the graph’s edges. In the second stage, GNNs is applied to predict the CVS, and an auxiliary reconstruction goal is introduced to fine-tune the rest of the model. Alapatt et al. (37) achieved end-to-end prediction of CVS. The ResNet-50 feature extractor was firstly pre-trained using Momentum Contrast. This contrast learning method learns image representations through minimizing the embedding differences between different augmented views of the same image and by maximizing the embedding distance between different images. Then initialize the classifier and fine-tuned based on the Endoscapes dataset to predict the CVS.

#### 4.1.3 Multi-task learning

**Figure 7:**
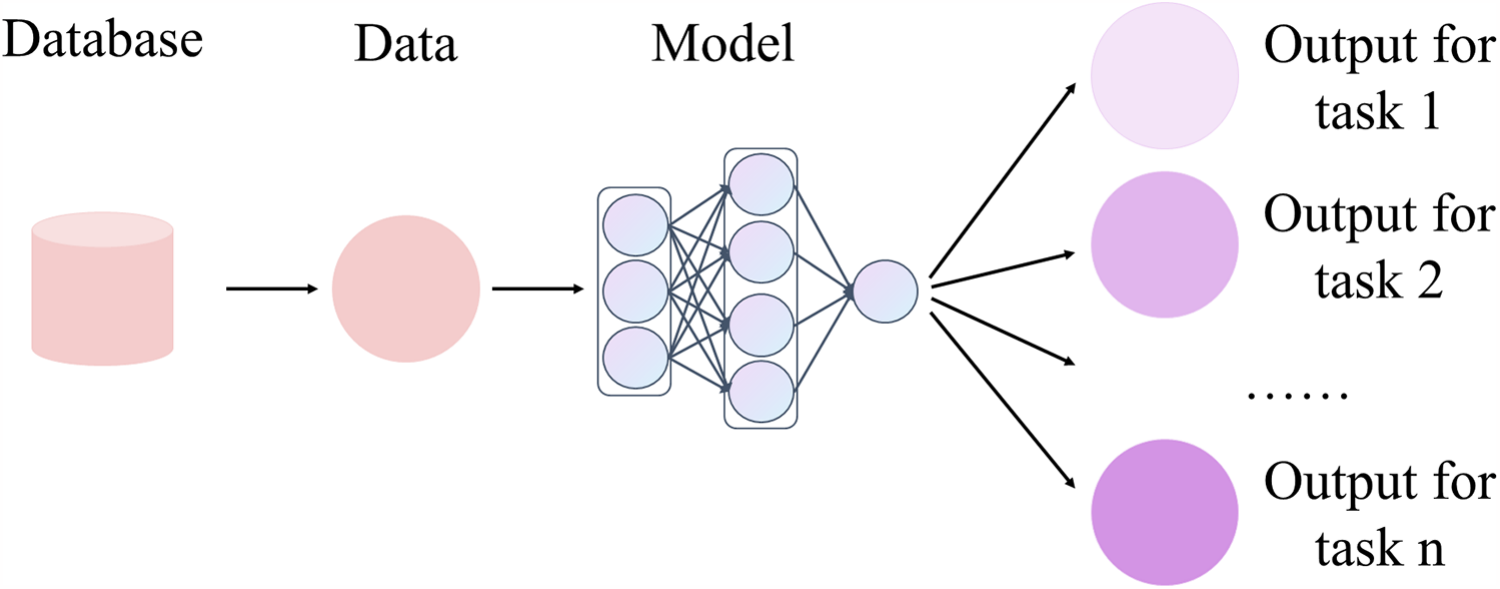
Frameowrk of multitask learning.

MTL aims to enhance the generalization performance of individual tasks and the overall model by learning multiple related tasks simultaneously. As displayed in Figure 7, in MTL, models are designed to address multiple tasks simultaneously rather than being trained independently for each task separately (57). These tasks usually show some correlation and thus can share the underlying feature representation, which can utilize the limited data resources in a more efficient way. Recent studies have shown that applying multi-task learning to surgical action recognition triad recognition can obviously improve the performance and robustness of the model.

Tripnet (29) is based on the MTL strategy and contains three branches including tool, verb, and target recognition. In this case, the class activation guidance unit is utilizing the weak localization information in the tool prediction, i.e., the class activation map, to guide the recognition of verbs and targets. And a new trainable 3D interaction space is proposed for capturing the associations between the triples components. However, there is still room for improvement in Tripnet’s fine-grained performance when dealing with complex scenes. To address this issue, Rendezvous (58) introduces two different attention mechanisms. One of them refers to the Class Activation-Guided Attention Mechanism based on Tool Activation Graphs, capturing spatial attention of the components of a single action triad in a scene for improved verb and target detection. The Multi-Headed Hybrid Attention module is a hybrid of self-attention and cross-attention, capturing the interaction and semantic relationships between tools, verbs, and targets.

To further enhance the extraction of spatio-temporal dependent features, the multi-task fine-grained spatio-temporal framework (MT-FiST) (59) was proposed by Li et al. The model contains four task branches for recognizing surgical tools, actions, targets, and triples. MT-FiST decouples global task features into category-aligned fine-grained features using a multi-labeled intercommunication channel loss function, which can enable the model to learn more local details in the surgical scene and ensure feature differentiation and diversity. And captures the temporal correlation between neighboring frames through a partially shared parameter LSTM cell. Yamlahi et al. (60) presented the first attempt to use self-distillation to address the problems of category imbalance and label ambiguity in surgical video analysis. At first, an instructor model is trained. Then, a student model is trained using soft labels to reduce the overconfidence problem. Second, a heterogeneous integration model is proposed, which can combine three different Swin Transformer sizes.

Recent studies have introduced GNNs that utilize graph structures to capture the complex relationships between elements during surgery. The MCIT-IG model (61) is consisted of two phases. In the first phase, embeddings for each target category are generated. In the second stage, a bidirectional dynamic graph is constructed to simulate the interaction between instrument instances and target category embeddings, and verbs are learned on the interaction edge features to detect triples. In addition, a hybrid supervised learning strategy that combines weak target presence labeling from MCIT and pseudo-triad labeling from IG is used to train the network. Concept Graph Neural Networks (32) incorporates knowledge graphs into surgical video analysis and models different concepts and their relationships during surgical procedures as graph structures. Elements of the surgical process (e.g., surgical tools, organization, etc.) can be modeled as nodes of the graph. Interactions between the elements are modeled as hyperedges of the graph.

#### 4.1.4 Evaluation metrics

Evaluation metrics are used to evaluate model performance and quantify how well a model performs in different dimensions, helping comprehend the strengths and weaknesses of a model. Totally ten categories of evaluation metrics were used in the classification methodology, each concentrating on a different evaluation dimension.

As shown in Figure 5, Acc and AP were used most frequently, 15 and 11 times, respectively. Acc evaluates the overall correct classification rate of the model, i.e., the number of correctly predicted samples as a proportion of the total number of samples. The high frequency of Acc usage may reflect the fact that overall correctness is still the most intuitive and popular evaluation criterion for a lot of tasks, especially when the class distribution is balanced. However, AP evaluates the balance between the Precision(Pre) and Recall(Rec) of the model under different thresholds, which is particularly appropriate for evaluating the performance of multi-class classification problems. The high-frequency use of AP suggests that researchers value the combined performance of the model under different confidence thresholds, particularly for applications in complex scenarios. The widespread use of Acc and AP may also be associated with their implementation and standardization in mainstream machine learning libraries, such as the support of these metrics in tools like scikit-learn, TensorFlow, and PyTorch.

Moreover, the other metrics were used relatively infrequently. In this study, pre and F1 scores were used 3 times each, and Rec was used 5 times. Besides, acc is simple and easy to understand but performs poorly on class-imbalanced datasets. Pre and Rec are suitable for class-imbalanced scenarios, especially when the cost of focusing on misclassification is high. Pre-high precision suggests fewer false positives but may miss them. Rec high recall indicates fewer misses but may miss them. F1 Score is used for balancing Pre and Rec, and is suitable for scenarios requiring a combination of the two. Krippendorff’s alpha (K-Alpha), Sensitivity (Sen), Specificity (Spe), and AUC Score were also used in specific applications, with each metric used once.

### 4.2 Segmentation methods

The current section explains the publications of segmentation methods, including 73 articles, as shown in Table 3. Firstly, they are grouped in line with learning strategies: supervised learning, semi-supervised learning, weakly supervised learning, and unsupervised learning. Subsequently, relevant studies based on transfer learning and attention mechanisms are shared. Finally, evaluation metrics of segmentation methods are analyzed.

**Table 3:** Segmentation methods.

References	Year	Learning strategy^a^	Target^c^	Model	Dataset^b^
(108)	2024	SL	Ureter, spleen,pancreas, etc.	SegFormer+Class Weight Adjustment	DSAD
(104)	2024	WSL	AW, liver, fat ,etc.	Segment Anything (SAM)+SegGPT	m2caiSeg, CholecSeg8k
(99)	2024	SSL	Liver etc.	Autoencoder+SegFormer	m2caiSeg, Cholec80, CholecSeg8k
(116)	2024	SL	Uretersm, nerves, etc.	Unet	Proprietary
(117)	2024	SL	Gallbladder, spleen, etc.	TernausNet++ TernausResNet+LinkNet+DeepLabV3	Proprietary
(105)	2024	SL	Hepatic vein, CBD, etc.	Encoder-Decoder+U-Net5ed+SegNet-VGG19+SegNet-VGG19+DeepLabv3+	CholecSeg8K
(118)	2024	SL	Uterus, ovaries, fallopian tubes, etc.	Densely Multi-scale Pyramid Module+Feature Fusion Module+Encoder-Decoder	Proprietary
(119)	2024	SL	Contours, bulges and ligaments	SAM+ResNet+DPE+SGA+BFU	Proprietary
(103)	2024	SL+USL	Liver etc.	DeepLab+HRNet32+HRNet48+Swin Transformer Small+Swin Transformer Base	CholecSeg8k
(97)	2024	SL	CVS	DeepCVS	Cholec80
(98)	2024	SL	Gallbladder, CBD, etc.	Channel Attention Pyramid Scene Parsing Plus Network: Pyramid Scene Parsing Plus Module+Multi-scale information Fusion Module Transfer learning	Cholec80
(120)	2024	SL	SMV, ICA, ICV etc.	DL model	Proprietary
(121)	2024	SL	Ureter	UreterNet+FPN	Proprietary
(106)	2024	SL	Liver etc.	SP-TCN+HRNetv2+Swin Transformer	CholecSeg8k
(101)	2024	SL	Endometriosis, CVS, etc.	DeepPyramid++PVF+DPR+VGG16+ResNet34+ResNet50	ITEC LapGyn4
(122)	2024	SL	Gallbladder, intestinal, etc.	Hierarchical Semantic Segmentation (HSS)+Hiera-Mix+Swin Seg	Proprietary
(54)	2024	SL	CVS	LG-CVS+Domain Generalization+Disentangled Learning+Latent Graph+GNNs	Endoscapes
(88)	2024	SL	Hepatic veins, glisson	Feature Pyramid Network(EfficientNetV2-L)+Transfer learning	Proprietary
(123)	2024	SL	Ureteral	Unet	Proprietary
(90)	2024	SL	11 anatomical structures	DeepLabV3, SegFormer+Attention+Multi-Teacher knowledge distillation+Integrated learning	DSAD
(19)	2024	SL	Liver, gallbladder, spleen, etc.	LDCNet: Res2Net+Attention+Encoder-Decoder+FCN	Proprietary
(79)	2024	SSL	Liver etc.	Multi-scale Projection Head	CholecSeg8k
(78)	2024	SSL	11 anatomical structures	DeepLabV3(ResNet50)	DSAD
(24)	2023	SL	16 different target structures	DeepLabv3(ResNet50)+Transfer learning	Proprietary
(124)	2023	SL	Left adrenal vein	ESFPNet+Transfer learning+Encoder-Decoder	Proprietary
(125)	2023	SL	Kidney	A GPU-based pixel-by pixel connectivity segmentation mechanism	Proprietary
(126)	2023	SL	Hepatic vein, glisson	DeepLabV3	Proprietary
(127)	2023	SL	Hepatic vein, glisson	DeepLabV3	Proprietary
(128)	2023	SL	Autonomic nerves	DeepLabV3+(Xception)+Transfer learning	Proprietary
(72)	2023	SL	Go/No-Go zones	GoNoGoNet	Proprietary
(65)	2023	SL	Uterine contour and tubal junction region	Unet	SurgAI3.8K
(20)	2023	SL	AW, colon etc.	DeepLabV3+SegFormer+Transfer learning+Attention	DSAD
(71)	2023	SL	Organs and tissues	LinkNet34+Decoder	CholecSeg8k
(84)	2023	USL		MoCo v2, SimCLR, DINO, SwAV	Endoscapes
(81)	2023	USL	AW, liver, fat, GB	Masked Frequency Consistency module+Segformer, DeepLabV2+Attention	CholecSeg8k
(40)	2023	SSL	CD, cystic artery	Unet+Multi-scale Projection Head+Auto-Encoder+Hybridloss function	Cholec80, M2cai16-tool, M2cai16-workflow
(62)	2022	SL	Areolar tissue	DeepLabv3+	Proprietary
(42)	2022	SL	Intestine	Unet, Vnet+Discriminator network	EndoVis 18’
(73)	2022	SL	Go/No-Go zones	GoNoGoNet	Proprietary
(76)	2022	SSL	Liver etc.	DeepLabv3+(ResNet)+Active learning	CholecSeg8k
(77)	2022	SSL	Liver, etc.	Unet(ResNet18)+K-Means	CholecSeg8k
(83)	2022	USL	CD, cystic artery	ResNet101+Label relaxation+Pseudo-label+FCN	Proprietary
(86)	2022	SL	mesenteric artery	DeepLabV3(ResNeSt-269)+Transfer learning	LapSig300
(87)	2022	SL	Organs	DeepLabV3+(ResNeSt)+UperNet(SwinTransformer)+Domain randomization	Proprietary
(63)	2022	SL	Uterus etc.	Mask R-CNN+YOLACT+YolactEdge	AutoLaparo
(66)	2022	SL	Liver	Unet	Proprietary
(67)	2022	SL	8 structures: AW, liver, fat etc.	UNet+UNet+++DynUNet+UNETR+DeepLabV3+	CholecSeg8k
(49)	2022	SL	CD etc.	DeepCVS: DeepLabV3+Multistage learning+Classification network	Proprietary
(23)	2022	SL	Loose connective tissue	SwinPA-Net: Swin Transformer+Dense Multiplicative Connection+Local Pyramid Attention	LIVis(Proprietary)
(39)	2022	SL	Go zone, No-Go zone, liver etc.	GoNoGoNet+CholeNet: ResNet50+Pyramid Scene Parsing Network+Multi-scale pyramid pooling	Cholec80; M2cai16-workflow
(21)	2021	SL	Loose connective tissue fibers	Unet+Data Augmentation	Proprietary
(129)	2021	SL	Liver etc.	Mask R-CNN	Proprietary
(130)	2021	SL	GI tract, blood, vessels, uterus etc.	CNNs	Proprietary
(35)	2021	SSL	CVS	TCNN: Multiloss learning+Auto-Encoder	Endoscapes
(82)	2021	USL	CD, cystic artery	ResNet101+Label relaxation+Pseudo-label	Proprietary
(70)	2020	SL	Blood vessels	NephCNN: Adversarial+training+FCNN	Nephrec9
(10)	2020	SL	Uterus, ovaries	Mask R-CNN+Transfer learning	SurgAI(Proprietary)
(102)	2020	SL	Liver, fat etc.	Unet+TernausNet+LinkNet+SegNet+FCN	EndoVis 19’
(69)	2020	SL	Occluding contours of the uterus	Unet+new loss function	Proprietary
(131)	2020	SL	Liver, GO zone, NO-GO zone etc.	CNNs	Proprietary
(75)	2020	SSL	Liver, gallbladder, intestine, artery etc.	CNNs+Encoder-Decoder+USL pretrain	m2caiSeg
(132)	2019	SL	Anatomical structures	Algorithm based on the extraction and matching of image features	Proprietary
(80)	2019	WSL	Liver, gastric etc.	DeepLabv3+FCN	Proprietary
(68)	2019	SL	Kidney	KiBo-Net(Unet)	EndoVis 17’ Kidney
(74)	2019	SL+SSL	Liver	Unet variant+Knowledge Distillation	Proprietary
(133)	2019	SL	Gallbladder, CD, bile duct	CNNs+Encoder-Decoder+Depthwise Separable Convolution+Flip-Based Subpixel Reconstruction	Proprietary
(134)	2019	SL	Organs	Xception+Encoder	Proprietary
(135)	2017	SL	Liver	Deep residual networks+FCN+Multi-resolution loss function	Proprietary
(136)	2016	SL	Organs	Superpixel Extraction+Descriptor+Classifier	MICCAI 2014
(137)	2016	SL	Liver, diaphragm, ligament and tissues	Intra-operative 3D Scene Reconstruction+Segementation using structures from Point Cloud+Labelling with laparoscopic scene cues	Proprietary
(138)	2015	SL	Uterus	Thresholding algorithm+SVM	Proprietary
(139)	2015	SL	Uterus	Gaussian Mixture Model	Proprietary
(140)	2014	SL	Uterus etc.	Gaussian Mixture Model	Proprietary

^a^For the “Learning Strategy” column. SL, supervised learning; WSL, weakly supervised learning.

^b^For the “Dataset” column, “Proprietary” means that the dataset is not publicly available.

^c^Target may involve surgical instruments, etc. Only anatomical structures are listed here.

#### 4.2.1 Supervised learning

The researchers used various deep-learning models and techniques to investigate the segmentation task. These models cover general-purpose semantic segmentation networks, including the DeepLabv3 family, Mask R-CNN, FPN, and models commonly used for biomedical image segmentation, including Unet and ESFPNet. The selection of models needs to consider their accuracy, speed, complexity, and performance on specific tasks.

The performance of DeepLabV3 in image semantic segmentation tasks has been extensively validated. The network employs techniques such as Dilated Convolution and Global Average Pooling to efficiently extend the receptive field and maintain detailed information, contributing to better capturing organ boundaries and fine texture information. Igaki et al. (62) performed semantic segmentation of sparse connective tissue by DeepLabV3+. AutoLaparo (63) used Mask R-CNN, YOLACT, and YolactEdge to segment anatomical structures, achieving good segmentation results.

When compared with the previous studies, the following articles focus on the application of networks such as Unet that are commonly used for biomedical image segmentation and are designed to be more specific to the characteristics of medical images and the needs of surgical tasks. Unet (64) is known for its unique encoder-decoder architecture and hopping connection design. A similar network architecture is usually used in segmentation tasks, as shown in Figure 6b. In this case, the encoder employs a representative structure of CNNs, which gradually extracts image features through multiple convolutional and pooling layers to map the input image to a low-resolution feature map. Different from traditional decoders, Unet’s decoder employs operations such as up-sampling and convolutional transposition to gradually restore the low-resolution feature map to the original input image’s size, helping refine the segmentation results by learning contextual information and detailed features. The jump connection in the Unet architecture connects the feature maps of each layer in the encoder to the corresponding decoder layer, which realizes cross-layer information transfer to better comprehend the semantic information of the image and improve the accuracy and robustness of segmentation.

SurgAI3.8K (65) is the first gynecological dataset with anatomical annotations, on which the authors employed the U-Net architecture to automatically perform the segmentation of the uterus, uterine contours, and regions of the left and right tubal junctions in surgical images. Bardozzo et al. (66) used a U-Net model for semantic segmentation of the liver, which was interpreted a posteriori by Grad-CAM and Grad-CAM++. Additionally, Silva et al. (67), compared the performance of different networks including Unet, Unet++, DynUNet, UNETR, and DeepLabV3+ on the CholecSeg8k dataset. The results demonstrate that the performance of different networks varies on segmentation tasks with different anatomical structures. This indicates that for a specific task, it is essential to consider the advantages and disadvantages of different models and choose the most suitable one.

Numerous studies have used U-Net or its variants as the basic segmentation network architecture, building on it with structural improvements or combining it with other approaches, such as adversarial training strategies, discriminator networks, or the incorporation of new loss functions.

For example, for the application in kidney edge detection, KiBo-Net (68) improves the structure of U-Net by adding additional convolutional and dropout layers. And the input of the network is modified to be the distance field, and by extracting the depth information and distance field of the image, it predicts whether the pixel belongs to the kidney edge or not. Franccois et al. (69) applied the U-Net architecture to detect the occluded contours of the uterus, proposed a new distance-based evaluation score, and enhanced the performance of the network by introducing a new structural penalty term. NephCNN (70) segment blood vessels in laparoscopic nephrectomy videos. The network utilizes a 3D fully convolutional neural network (FCNN) as a segmenter to extract spatio-temporal features and enhance temporal continuity between pixels. An adversarial training strategy is employed to maintain the coherence of the vessel shape by constraining the segmentation results through a discriminator network. The experimental results of NephCNN significantly outperform 2D U-Net and 3D U-Net.

When dealing with small datasets, Monasterio et al. (42) used U-Net and V-Net as segmentation networks, first synthesizing the erroneous segmentation labels and training a discriminator network to detect errors and produce a dense segmentation error map. Subsequently, the segmentation network is co-trained by minimizing the discriminator prediction error with the standard segmentation loss. Uramoto et al. (71) introduced a second-level decoder on top of the base U-Net structure, which adds semantically similar group segmentation of images as a second-level task. The feature maps of the second-level decoder are also fused into the first-level decoder to enrich the latter’s feature representation. The approach achieves better performance.

With the development of DL techniques, more researchers have begun to explore alternative encoder-decoder architectures other than Unet. These improved approaches enhance the model’s feature extraction and reconstruction capabilities by introducing new modules and techniques, such as feature pyramid networks and multi-scale feature fusion.

GoNoGoNet (39) is applied to recognize anatomical structures, safe areas, and dangerous areas in LC. The network combines ResNet50 and the pyramid scene parsing network. The pyramid pooling module aggregates feature maps from ResNet50 at four different scales. Afterwards, it is mapped to pixel-level classification scores through a convolutional layer. The whole process can be regarded as an encoder-decoder model. Both Khalid et al. (72) and Laplante et al. (73) evaluated the GoNoGoNet model. The results showed that GoNoGoNet accurately identified safe and dangerous zones in the LC.

#### 4.2.2 Semi-supervised learning

The core idea of semi-supervised learning(SSL) is to combine a limited amount of labeled data with a large amount of unlabeled data in order to improve model performance. Next, the application of semi-supervised learning strategies is explored to segmentation tasks.

Fu et al. (74) compared the performance of supervised and semi-supervised learning methods. A combination of supervised and unsupervised loss and an exponential moving average updating strategy for the teacher network is demonstrated through a semi-supervised mean teacher training paradigm. Higher segmentation accuracy and stability are demonstrated compared with the Unet-based supervised network.

However, the issue of insufficient labeled data remains a challenge. To address this, m2caiSeg (75) employed unsupervised pre-training and data augmentation techniques. Despite the good performance on some categories, there is still room for the improvement of the performance for rare categories. To further enhance performance, TCNN (35) utilizes spatially and temporally supervised signals provided by a self-encoder network incorporating temporal cues and anatomical constraints. The framework demonstrates how a low-dimensional representation of the prediction mask can improve performance while maintaining low computational costs.

Recognizing the need for more effective sample selection for labeling, Qiu et al. (76) introduced an active learning method called class-level confidence-aware active learning. The method selects the most informative samples by keeping a class-level confidence bank and combining the confidence scores. The method can achieve better segmentation with a limited labeling budget through effectively utilizing the unlabeled dataset. Similarly, ALGES (77) is also an active learning method selecting the most representative and diverse samples by calculating the predictive segmentation of unlabeled images and the gradient of the model parameters, reducing the labeling workload and improving the model performance.

To maximize the use of existing labels, Jenke et al. (78) trained a surgical scene segmentation model by combining multiple partially annotated datasets. This method incorporated supplemental annotations during model training, significantly improving DC scores and reducing confusion between categories. In addition, Zhang et al. (79) proposed a class-level contrast learning method that introduces a multi-scale projection header and improves the partitioning of the positive sample pairs to learn the contrast of the extracted features at each scale. The model is trained using both segmentation and classification labels. Even though only a relatively small number of labels (1%–10%) exhibit high intersection-unification (IoU) scores.

#### 4.2.3 Weakly supervised learning

Weakly Supervised Learning (WSL) is also one of the vital methods that can be used to address the challenge of labeling laparoscopic images. In SL, the training data is labeled with exact labels, providing clear guidance for the model. In semi-supervised learning (SSL), the model is trained on a small portion of labeled data combined with a larger amount of unlabeled data to leverage both types of information. Meanwhile, in USL, the training data is entirely unlabeled, requiring the model to detect patterns without any label guidance. WSL, however, is characterized by training data with partially accurate or incomplete labels, offering a flexible solution when precise labeling is challenging or costly.

Fuentes et al. (80) proposed a novel method for labeling laparoscopic images, using “stripes” as a weak annotation and combining it with a partial cross-entropy loss function to train a FCNN for scene segmentation. According to experimental results, the segmentation accuracy of the method is close to that of a fully supervised method on three different datasets, while the time required for labeling is reduced by approximately 13 times.

#### 4.2.4 Unsupervised learning

Unsupervised learning (USL) is an approach in machine learning which is opposed to supervised learning. In unsupervised learning, the training data does not contain labels or results. Meanwhile, the algorithm needs to figure out the hidden patterns and structures in the data itself. Next, we will delve into how unsupervised learning can be used for segmentation tasks.

The Masked Frequency Consistency (MFC) module (81) is employed to solve the problem of domain adaptive semantic segmentation of laparoscopic images. The module is implemented by image frequency representation, masking strategy and consistency regularization. The MFC method is demonstrated to be comparable to fully supervised methods without manual annotation, facilitating knowledge transfer from computer simulations to real laparoscopic datasets as well as enabling model generalization across domains.

Next, the following study explores self-supervised learning, an approach that utilizes the structure and intrinsic relationships of the data itself for feature learning. Owen et al. (82, 83) applied both label relaxation and pseudo-label self-supervision strategies. The label relaxation method will transform the traditional segmentation problem into a heat map regression problem, where the true label heat map is obtained based on the Euclidean distance transform of the original annotation. Moreover, and this method is capable of better dealing with the fuzzy labels of the structure in the image. At first, the pseudo-labeled self-supervised curation method trains a new model in the teacher-student architecture by training an initial model on labeled data and then using the predictions of that model as pseudo-labels for unlabeled data.

Among them, the article Detection (82) is based on FCN and uses ResNet101 as the backbone network. Besides, cross-entropy loss and soft cross-entropy loss are employed to train the model. The method achieved high accuracy and was recognized by surgeons. Instead, Alkhamaiseh et al. (40) combined unsupervised pre-training and supervised fine-tuning. In this, the autoencoder extracts features from a partially prepared dataset and uses these features as pre-training weights for the U-Net encoder layer. Finally, Ramesh et al. (84) evaluated the performance of four state-of-the-art SSL methods including MoCo v2, SimCLR, DINO, and SwAV on a surgical video dataset and also investigated the SSL methods regarding different hyper-parameter settings, data availability, and generalization capabilities, exhibiting their potential in dealing with small datasets and data-scarce domains.

#### 4.2.5 Transfer learning

**Figure 8:**
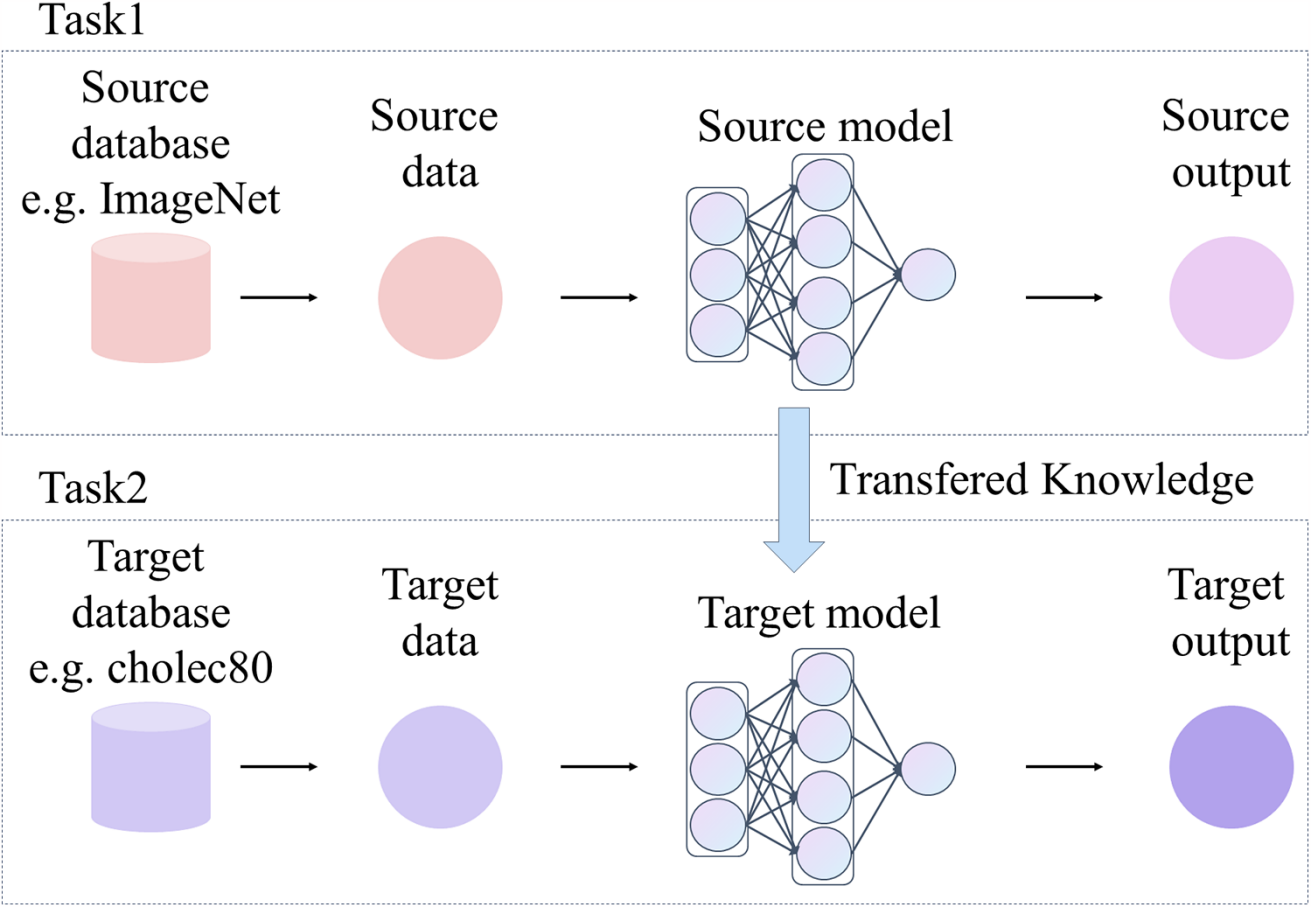
Frameowrk of transfer learning.

Transfer learning is a technique widely used in DL, as shown in Figure 8, which can migrate the learned knowledge to a specific task by using models pre-trained on large datasets (e.g., ImageNet). This approach significantly reduces the training time and improves the model’s performance in tasks with limited data and performs particularly well in medical image analysis (85). Laparoscopic surgical images suffer from problems including limited data volume and labeling difficulties. This makes it challenging to train models from scratch. In this case, transfer learning provides an efficient solution. That is, the pre-trained model is fine-tuned on surgical images to adapt it to a specific application scenario.

DeepLabV3 is fully pre-trained on large-scale datasets such as ImageNet. Meanwhile, DeepLabV3 features a flexible architecture that can be built based on different backbone networks (e.g., ResNet, ResNeSt, Xception, and EfficientNet.). Therefore, researchers can choose a suitable model according to the demands of specific tasks and the limitations of computational resources. DeepLabV3 and its variants are implemented in numerous open-source frameworks, such as TensorFlow, and PyTorch, providing rich tools and documentation. On this basis, DeepLabv3 becomes a widely used pre-training model for segmentation tasks.

Kitaguchi et al. (86) used DeepLabv3+ as a backbone for migration learning and training on LapSig300, showing that the model was able to accurately recognize IMAs at over 12 FPS with an mDC of 0.798. The feasibility for real-time navigation of blood vessels in laparoscopic colorectal surgery was demonstrated. Next, Yoon et al. (87) proposed semantic image synthesis using a virtual surgical environment to enhance the performance of surgical scene segmentation. Through the combination of manual synthetic data, domain randomized synthetic data with real data, which provides a wider dataset. The results show that synthetic data can significantly enhance the performance of the models on low-performance categories.

In addition to deeplabv3, networks such as Mask R-CNN can be used as pre-trained models to provide powerful feature extraction for segmentation tasks.

Madad et al. (10) achieved accurate localization and segmentation of key structures such as the uterus, ovaries, and surgical tools by adopting Mask R-CNN for migration learning. Une et al. (88) developed models for identifying the hepatic veins and glissonean based on a feature pyramid network (FPN) with EfficientNetV2-L as the backbone. The results showed high accuracy and sufficient processing speed.

#### 4.2.6 Attention methods

**Figure 9:**
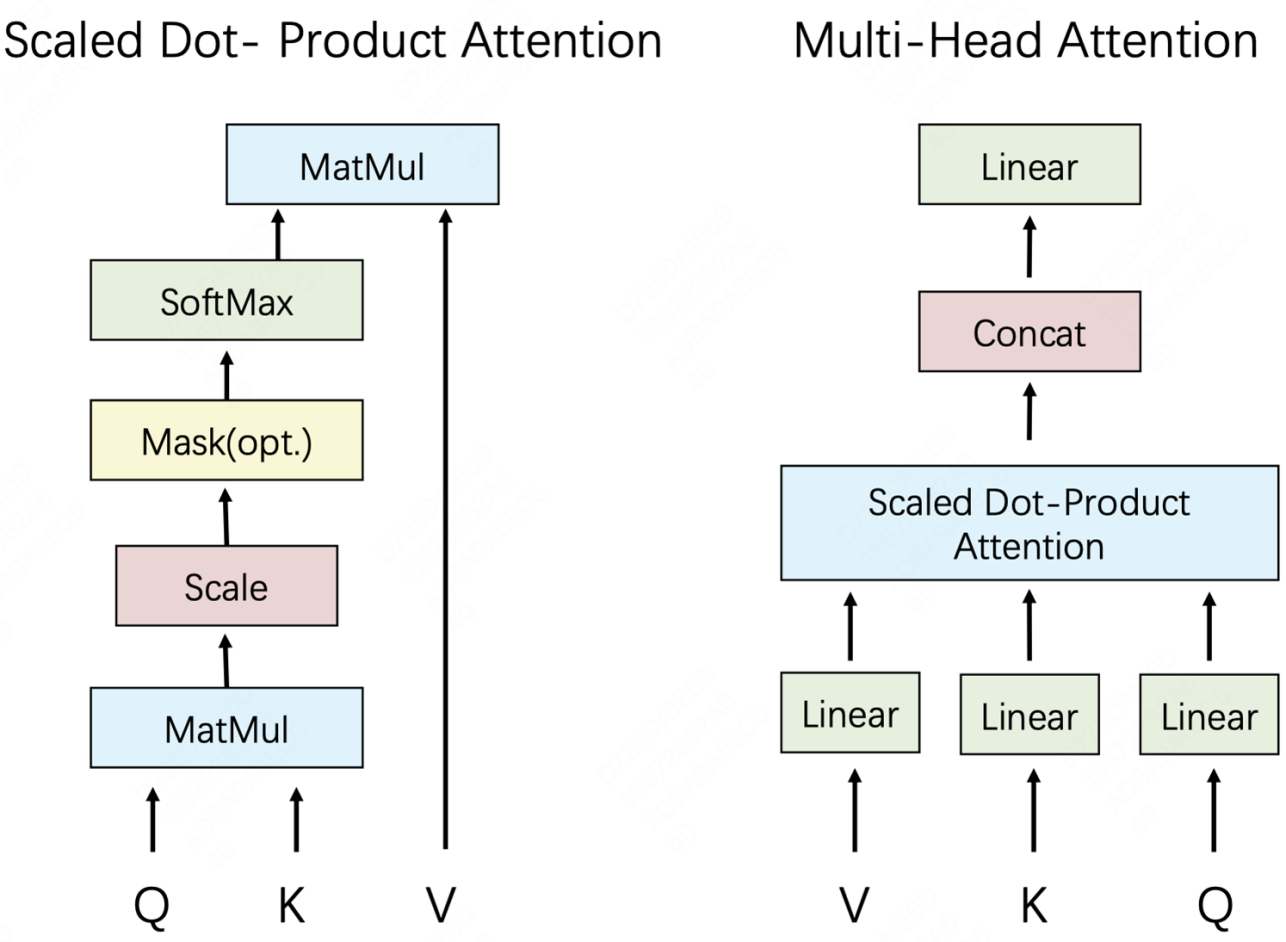
(left) Scaled dot-product attention. (right) Multi-head attention.

Attention Mechanism enhances the network’s ability to focus on specific parts during information processing, similar to the human visual process: when processing a large amount of information, more attention is paid to what is relevant to the task, thus enhancing the efficiency of information utilization. In neural networks, the attention mechanism is implemented by assigning different weights to input features, i.e., determining the region that the model should focus on based on the correlation between input elements (89). As shown in Figure 9, a commonly used implementation is the dot-product attention mechanism, which measures the relevance of elements by calculating the dot product between query vectors and key vectors, and then generates the attention weights to weight the value vectors so as to focus on key regions. In addition, to enhance the model’s representation of different features, the Multi-Head Attention mechanism applies multiple attention heads in parallel and computes the attention independently in different projection spaces, enabling the model to capture information from multiple perspectives. This mechanism has great adaptability and flexibility in dealing with complex data, and is especially suitable for dealing with laparoscopic surgical images with complex and unevenly distributed structures, which can still effectively focus on the key parts when the anatomical structures are deformed and distorted, thus improving the accuracy of the network.

SwinPA-Net (23) used the Swin Transformer as an encoder. Second, efficient information transfer between feature hierarchies can be achieved through dense multiplicative connections. In the meanwhile, the local pyramid attention module helps the network better focus on key regions and aggregate multi-scale features. On the decoder side, a gradual upsampling strategy is applied to maintain the fineness of the segmentation results. SwinPA-Net achieves state-of-the-art performance in three medical image segmentation tasks. Kolbinger et al. (20) trained independent and joint models based on DeepLabv3 and SegFormer for 11 anatomical structures in laparoscopic images. SegFormer’s self-attention mechanism can model the dependencies between different locations. Experimental results indicate that the models based on the attention mechanism have higher segmentation accuracy and generalization ability compared to those using only CNNs.

In practical applications, real-time is also an important consideration. To this end, Maack et al. (90) proposed an approach based on multi-teacher knowledge distillation. The mDC score of the real-time network is improved by training multiple DeepLabv3 and SegFormer-based teacher networks and aligning the outputs of these teacher networks with the output of a student network. Lightweight dynamic convolution network (LDCNet) (19) is a lightweight novel dynamic convolution network for real-time segmentation. A dynamic convolution-based attention module is used between the coders and decoders, allowing the network to better focus on regions of interest. The encoder part uses Res2Net and introduces a sensory wild block module to further refine the features. The progressive decoder obtains effective feature reconstruction by fusing high-level features with low-level features. The experimental results suggest that LDCNet combines high speed and accuracy and exhibits high practical value in real scenarios.

#### 4.2.7 Evaluation metrics

Thirteen different evaluation metrics were used in the segmentation task, with significant differences in their frequency of use.

As presented in Figure 5c, the Dice Coefficient (DC) is one of the most commonly used segmentation metrics to measure the similarity between predicted segmentation and true segmentation. DC was used for totally 27 times in the literature, suggesting that researchers place great importance on the quality of the overlap of segmented regions. IoU is another widely used metric, which was used a total of 24 times, aiming to assess the intersection and concurrency ratio between predicted segmentation and true segmentation.

Acc, F1 Score, Rec, and Pre also have some applications in the segmentation task. Rec measures the model’s ability to detect all positive sample regions in the segmentation task, focusing on regions that are not correctly segmented. Pre evaluates the proportion of positive sample regions predicted by the model that are actually positive, emphasizing the accuracy of the prediction. Acc is of comparatively low importance because the segmentation task is more concerned with regions of overlap and matching.

Other metrics, including Sen, correlation, agreement are used less frequently. AP is used in some complex multi-class segmentation tasks. Hausdorff_95 evaluates the maximum distance between the predicted segmentation and the true segmentation boundary, and is mainly applied in segmentation tasks requiring high accuracy. Mean Misrecognition measures the missegmentation region of the model. Error evaluates the overall segmentation error of the model.

### 4.3 Object detection methods

In the current section, we will discuss the research associated with the target detection task, which consists of 15 articles, as shown in Table 4. These studies can be categorized into two main groups: one is based on traditional image processing algorithms, and the other is based on DL methods. Next, we will elaborate on these two directions, first presenting the research progress of traditional image processing algorithms and then discussing the latest results based on DL.

**Table 4:** Object detection methods.

References	Year	Learning strategy^a^	Target^c^	Method	Dataset^b^
(141)	2024	SL	Liver base of segment IV,Rouviere’s sulcus	YOLOv7	Proprietary
(142)	2024	SL	Vas deferens and subabdominal vessels, etc.	YOLOv8	Proprietary
(36)	2023	WSL	Gallbladder, CD, cystic artery, hepatic duct, cystic plate, etc.	LatentGraph-CVS: constructing graphical representations containing semantic information about anatomical structures and visual features+GNNs	Endoscapes
(26)	2023	SL	LM-RS, LM-S4, LM-CBD, LM-CD	YoloV3	Proprietary
(94)	2023	SL	CBD, CD, S4, RS	YoloV3	Proprietary
(95)	2023	SL	EHBD, CD, S4, RS	YoloV3	Proprietary
(25)	2023	SL	Uterus	Comparing the performance of Faster R-CNN, SSD, CenterNet, EfficientDet, YOLOv4, YOLOv5, YOLOv7+transfer learning	Proprietary
(18)	2022	SL	Endometriosis	Faster R-CNN(ResNet50), Mask R-CNN(ResNet101)	GLENDA
(143)	2022	SL	CD, cystic artery, CBD, cystic plate	SurgSmart: YoloV3(ResNet)+transfer learning	Proprietary
(144)	2021	SL	CD, CBD, S4, RS	YoloV3	Proprietary
(145)	2021	SL	Anatomical landmarks	Yolo,YoloV4 tiny	Proprietary
(27)	2021	SL	Vas deferens	YoloV4	Proprietary
(93)	2021	SL	Endometriosis	Conventional image processing techniques	GLENDA
(92)	2017	SL	Triangular tissue	Conventional processing techniques	Proprietary
(91)	2015	SL	FU-junctions	Conventional image processing techniques	Proprietary

^a^For the “Learning Strategy” column. SL, supervised learning; WSL, weakly supervised learning.

^b^For the “Dataset” column, “Proprietary” means that the dataset is not publicly available.

^c^Target may involve surgical instruments, etc. Only anatomical structures are listed here.

#### 4.3.1 Based on conventional image processing techniques

The next presentation is a study based on traditional image processing techniques adopted for addressing tasks, including lesion detection. The core idea of traditional image processing techniques is to obtain the localization and identification of the target of interest through mathematical manipulation and feature analysis, mainly including feature extraction and pattern recognition.

Prokopetc et al. (91) trained uterus detectors and FU connection detectors. These detectors incorporate connection-specific context-sensitive features to achieve automatic target detection through linear classification. Nakasuji et al. (92) successfully identified triangular tissue regions of pulled by surgical forceps, by combining corner point detection and ridge detection with Delaunay triangular dissection. Visalaxi et al. (93) extracted focal regions of endometriosis using OpenCV, adaptive thresholding, and contour masking, and evaluated the recognition effect by the mean intensity value.

#### 4.3.2 Based on deep learning

The aforementioned methods based on traditional image processing techniques have been successful to a certain extent, while they have many limitations, such as the comparative sensitivity to image quality and parameter selection, as well as the lack of precision in localizing specific targets. Therefore, the subsequent research incorporated novel techniques, aiming to achieve higher performance.

The DL models in these articles cover a wide range of classical target detection algorithms, such as YOLOv3, and Faster R-CNN. Mask R-CNN employs a two-step detection process. First, a pre-trained ResNet backbone network is used to pull out features. Afterwards, the features are sent through a feature pyramid network for fusion. Then, more Region Proposal Networks (RPNs) propose bounding boxes. RoI Pooling and RoI Align pull out features and perform target detection or pixel-level segmentation. A similar network architecture is usually used in target detection tasks, as shown in Figure 6c. By contrast, the YOLO family of models excels in real-time applications with its high inference speed and good detection performance for scenarios requiring fast processing.

Nakanuma et al. (26) developed a YOLOv3-based AI system for the detection of anatomical marker points that surgeons rely on during surgery. The system’s performance was evaluated by an external evaluation committee, showing that the system could accurately identify key anatomical landmarks. Similarly, Fujinaga et al. (94) and Endo et al. (95) conducted a similar study. The results demonstrated the method’s effectiveness. In addition to the direct application of YOLOv3, boonkong et al. (25) compared the performance of eight models on uterine detection tasks, including Faster R-CNN, SSD, CenterNet, EfficientDet, YOLOv4, YOLOv5, and YOLOv7. Based on the obtained results, YOLO series models perform optimally in terms of accuracy and speed.

#### 4.3.3 Evaluation metrics

As shown in Figure 5, target detection methods use a wide variety of evaluation metrics, including both qualitative and quantitative evaluations.

Quantitative evaluation is based on numerical computation and image analysis and is mainly adopted for measuring the performance of algorithms in terms of localization and accuracy. Among them, AP is used most frequently. There are some qualitative evaluation methods, such as five-level scale evaluation and questionnaire format evaluation. Moreover, the effectiveness of the algorithm is evaluated from the point of view of user experience and practical application by collecting users’ subjective feelings and opinions on the effectiveness of the algorithm.

## 5 Discussion

### 5.1 Datasets

Totally 24 publicly available datasets were involved in the study of anatomical structures in laparoscopic surgical images, among which 7 were generalized datasets and 11 were proprietary datasets. The most frequently used dataset was the generic dataset, which was used for 13 times. Although they were used more frequently, their additional annotation work remained a challenge for research efficiency and resource allocation.

Aside from the high-frequent use of the generic datasets, there was a large variation in the frequency of application of the other datasets, with 15 datasets being used only once. This situation reveals the dispersion and diversity of dataset use within the field, possibly caused by the diversity of research topics and the unique strengths of specific datasets. This diversity allows researchers to select the most appropriate datasets for different problems, driving innovation in methods and techniques. However, this decentralization also brings certain disadvantages that may result in duplication of effort: researchers need to develop or adapt specific processing methods for each new dataset, which can thus increase research time and costs. Secondly, the lack of extensive validation and application of datasets used at low frequencies may hide some undiscovered flaws or limitations, lowering the reliability of the research results.

The comparison of similar tasks reveals that they all employ different datasets. This makes it difficult to directly compare the methods and results of different studies, limits the unified assessment and standardization of techniques in the field, and increases the complexity of comprehensively assessing the effects of different methods.

It is of note that most of the datasets contain annotations not only for anatomical structures but also for tools, surgical stages, and maneuvers. The multiple-annotated datasets provide rich contextual information for research and help develop more comprehensive and intelligent algorithms to enhance the adaptability and accuracy of models in real surgical settings. Additionally, seven datasets were generated from competitions or other events organized by MICCAI. These competition datasets drive the development of technology and innovation in the field through providing a standardized evaluation platform.

Finally, it is vital to emphasize that more than 60 articles used private datasets. Their experimental results are difficult to compare with other methods. More importantly, because private datasets are difficult to access, this can limit the possibility for other research groups to carry out further studies on the data, also resulting in unrepeatable and unvalidated experimental results.

To counter the existing problems, more collaboration and sharing of resources may be needed to improve the overall efficiency of research and the credibility of results. Therefore, we call on future researchers to actively use publicly accessible and comparable datasets to develop and validate their methods, as well as to openly collect and self-label their data so that other studies can access and use these datasets.

### 5.2 Methodology

Regarding methodology, each category of tasks is characterized by its own research methods and trends.

Classification tasks are mainly categorized into supervised learning and unsupervised learning methods. Supervised learning methods dominate the classification task, usually by fine-tuning the DL model or utilizing data enhancement techniques to improve the model performance. Weakly supervised learning and unsupervised learning methods mainly focus on the CVS prediction task. Multi-task learning methods are widely applied in surgical action triplet recognition tasks. These methods perform well when handling complex tasks, while for some simpler classification tasks, simple transfer learning has been capable of achieving good results.

The vast majority of studies in segmentation tasks depend on manually labeled data, and the use of supervised learning methods is the most common strategy. In supervised learning, transfer learning is widely applied. Meanwhile, attention mechanisms have also become a hot research direction, exhibiting the potential to improve segmentation accuracy. However, due to the high cost of manually labeling data, semi-supervised learning, weakly supervised learning, and unsupervised learning methods are also gradually gaining attention and application as a trend for future development. Graph neural networks are beginning to show promising applications in segmentation tasks, providing novel solutions.

There is relatively little literature related to the target detection task, and the early research is mainly based on traditional image processing techniques, while the research in the last five years is mainly based on DL methods. Most methods directly apply the existing model or are fine-tuned by transfer learning. However, the field of target detection has not yet been significantly developed, and there remains more room for innovation and improvement.

The development of these methods and techniques has brought significant progress and wide application prospects in the field. Nevertheless, there are also a lot of challenges and opportunities. Supervised learning methods, despite their superior performance, rely on a large amount of manually labeled data, and data acquisition and labeling are costly. Although research and application of unsupervised and weakly supervised learning methods can alleviate this problem to a certain extent, their accuracy and stability still need to be addressed. Real-time is another vital challenge, especially in high-risk environments such as surgery, where the inference speed of algorithms directly influences clinical decisions and patient safety. However, only a small portion of the literature focuses on inference speed, and future research needs to focus on the real-time optimization of algorithms to satisfy the needs of clinical applications. And with the continuous development of new technologies, including MTL, attention mechanisms, and GNNs, the performance and application scope of DL methods will be further improved. Moreover, this provides researchers with a wealth of research topics and innovation space, bringing new opportunities for progress in the field.

In addition, large models have achieved introduced attention in various domains, however, in this particular domain, they are limited to a few applications in categorization tasks, but all of them perform well. This demonstrates the great potential and necessity of exploring VLM. The multimodal fusion capabilities of VLM are leveraged in order to enhance the understanding of complex anatomical structures in laparoscopic surgical images. In addition, their strong generalization capabilities and ability to handle data scarcity make them ideal for dealing with high annotation costs and restricted data volumes.

### 5.3 Evaluation metrics

Evaluation metrics exert a vital role, not only in assessing algorithm performance, but also in directly influencing the application and diffusion of algorithms in clinical practice.

Current statistics find that classification, segmentation, and target detection tasks all involve multiple types of evaluation metrics. There are fewer cases where the tasks are the same and the evaluation metrics are also the same, making it difficult to comprehensively assess and compare the performance of different algorithms. Secondly, in the target detection domain, the number of evaluation metrics is comparable to that of the segmentation task, even though the number of literatures involved is relatively small. This maysuggest that the target detection domain has not been well addressed for the harmonization of evaluation metrics. In summary, we call for the use of more consistent and comprehensive evaluation metrics, aiming to more intuitively assess and compare the performance of different algorithms and to promote further development in the field.

In addition, we note that the target detection task involves qualitative evaluation. This suggests that in addition to quantitative evaluation metrics, it is increasingly vital to consider the actual usage and experience of physicians. Therefore, we suggest including more qualitative evaluation metrics in order to comprehensively assess the applicability and usefulness of the algorithms in a clinical setting. As a result, when selecting evaluation metrics, their correlation with clinical outcomes should be considered to ensure that the model can contribute in practical clinical applications.

### 5.4 Summary of challenges and potential future work

According to the literature we have collected, research in this area has shown a trend of rapid growth from year to year, showing widespread interest and sustained investment of resources.

In terms of datasets, the large number and diversity of types lead to problems of fragmentation and duplication in the use of datasets. While this diversity drives innovation in techniques and methods, it can also increase research time and costs, making it difficult to directly compare the results of different studies. In addition, numerous studies use private datasets, making it difficult to reproduce and validate results. Future studies should encourage researchers to use publicly available and comparable datasets, or to make publicly available self-collected and labeled data, hoping to increase the transparency and reproducibility of studies.

In terms of methodology, different tasks are characterized by different research methods and trends. Classification tasks mainly depend on supervised learning, but weakly supervised learning and unsupervised learning are also gaining attention, especially in CVS prediction tasks. Supervised learning methods are most commonly used in segmentation tasks, but semi-supervised, weakly supervised and unsupervised learning methods are also trending due to the high cost of data labeling. There is less literature on target detection tasks, and not yet significantly developed. Moreover, the application of VLM is relatively small, and future research should attempt to combine it with image analysis of laparoscopic surgery, which is expected to significantly improve the technical level and practical application in this field.

In terms of evaluation metrics, there is a wide variety of evaluation metrics involved in classification, segmentation and target detection tasks. In addition, future research should advocate the use of more consistent and comprehensive evaluation metrics to more intuitively assess and compare the performance of different algorithms. Meanwhile, qualitative evaluation metrics should be added to take into account the actual use and experience of physicians and to establish a correlation with clinical outcomes to ensure the effectiveness of the model in practical applications.

The continued growth and technological innovations in this field of research have exerted a profound impact on clinical practice. As a growing body of studies focus on solving real-world surgical challenges, we can anticipate the emergence of more accurate and smarter surgical assistance systems and tools in future clinical practice. These systems and tools will significantly improve the precision and safety of surgery, reduce complications and surgical risks, and therefore, provide better outcomes and quality of life for patients.

## 6 Conclusion

This study provided an overview of recent developments in the field of classification, segmentation and target detection of anatomical structures in laparoscopic images. The core subtasks and their applications in real medical scenarios were first discussed, followed by a statistical analysis of the current state of use of the dataset. Then, the methods, models, and evaluation metrics used in the literature were thoroughly analyzed and discussed, offering insights and reflections on current research. Finally, directions and strategies for future development were proposed to address the existing shortcomings and challenges, hoping to foster further development and innovation in the field.
